# Structure-Activity Relationship of Synthetic 2-Phenylnaphthalenes with Hydroxyl Groups that Inhibit Proliferation and Induce Apoptosis of MCF-7 Cancer Cells

**DOI:** 10.1371/journal.pone.0141184

**Published:** 2015-10-22

**Authors:** Chi-Fen Chang, Ci-Yi Ke, Yang-Chang Wu, Ta-Hsien Chuang

**Affiliations:** 1 Department of Anatomy, School of Medicine, China Medical University, Taichung, Taiwan; 2 Department of Medical Laboratory Science and Biotechnology, China Medical University, Taichung, Taiwan; 3 School of Pharmacy, China Medical University, Taichung, Taiwan; 4 Research Center for Chinese Herbal Medicine, China Medical University, Taichung, Taiwan; Institute of Biochemistry and Biotechnology, TAIWAN

## Abstract

In this study, six 2-phenylnaphthalenes with hydroxyl groups were synthesized in high yields by the demethylation of the corresponding methoxy-2-phenylnaphthalenes, and one 2-phenylnaphthalene with an amino group was obtained by hydrogenation. All of the 2-phenylnaphthalene derivatives were evaluated for cytotoxicity, and the structure-activity relationship (SAR) against human breast cancer (MCF-7) cells was also determined. The SAR results revealed that cytotoxicity was markedly promoted by the hydroxyl group at the C-7 position of the naphthalene ring. The introduction of hydroxyl groups at the C-6 position of the naphthalene ring and the C-4**'** position of the phenyl ring fairly enhanced cytotoxicity, but the introduction of a hydroxyl group at the C-3**'** position of the phenyl ring slightly decreased cytotoxicity. Overall, 6,7-dihydroxy-2-(4'-hydroxyphenyl)naphthalene (**PNAP**-**6h**) exhibited the best cytotoxicity, with an IC_50_ value of 4.8 μM against the MCF-7 cell line, and showed low toxicity toward normal human mammary epithelial cells (MCF-10A). **PNAP**-**6h** led to cell arrest at the S phase, most likely due to increasing levels of p21 and p27 and decreasing levels of cyclin D1, CDK4, cyclin E, and CDK2. In addition, **PNAP**-**6h** decreased CDK1 and cyclin B1 expression, most likely leading to G_2_/M arrest, and induced morphological changes, such as nuclear shrinkage, nuclear fragmentation, and nuclear hypercondensation, as observed by Hoechst 33342 staining. **PNAP**-**6h** induced apoptosis, most likely by the promotion of Fas expression, increased PARP activity, caspase-7, caspase-8, and caspase-9 expression, the Bax/Bcl-2 ratio, and the phosphorylation of p38, and decreased the phosphorylation of ERK. This study provides the first demonstration of the cytotoxicity of PNAPs against MCF-7 cells and elucidates the mechanism underlying PNAP-induced cytotoxicity.

## Introduction

Breast cancer is the most common cause of cancer death in females; therefore, the search for a new and effective anticancer agent is imperative. Naphthalene derivatives display potent anti-arrhythmia, anti-tumor, and antioxidant activities [1]. Pharmacologically, 2-phenylnaphthalenes (PNAPs) have similar spatial and conformational requirements to genistein (an isoflavone) and exhibit a large number of biological and biomedical effects [2]. Chemically, 1-substituted naphthalene, not 2-substituted naphthalene, is obtained by the electrophilic aromatic substitution of naphthalene. To the best of our knowledge, studies on the relationship between the structure and cytotoxicity of multi-substituted PNAPs are rare. In this study, we synthesized unsubstituted **PNAP**-**1**, seven methoxy-PNAPs (**PNAP**-**2**−**PNAP**-**8**), six corresponding hydroxy-PNAPs (**PNAP**-**2h**−**PNAP**-**7h**), and one amino-PNAP (**PNAP**-**8h**) and investigated their anticancer structure-activity relationships and mechanisms of action in the MCF-7 cell line.

Several studies have demonstrated that genistein and compounds with the phenyl-1-benzopyran-4-one backbone inhibit the growth of cancer cells via cell cycle arrest and the induction of apoptosis [3–5]. Cyclin-cyclin dependent kinase (CDK) complexes are the principal regulators of the cell cycle, which is a very complex and tightly regulated process [6,7]. The G_1_/S phase transition is regulated by activation of the cyclin D1-CDK4/6 complex and the cyclin E-CDK2 complex [8,9], whereas the G_2_/M phase transition is regulated by activation of the cyclin B1-CDK1 complex [10,11]. Deregulation of the cell cycle causes a lack of differentiation and aberrant growth.

Apoptosis is an evolutionary process that leads to programmed cell death [12]. The process of apoptosis includes morphological changes (e.g., cell shrinkage, membrane blebbing, chromatin condensation, and nuclear fragmentation) and biochemical changes (e.g., DNA breakdown, protein cleavage, and protein cross-linking) [13,14]. Many anticancer agents induce cell death by activating caspases, which form part of a common apoptotic pathway [15,16]. Extrinsic and intrinsic pathways that regulate caspase-dependent apoptosis have been identified [17]. In the extrinsic pathway, Fas, a death receptor, leads to the formation of a death-inducing FADD-caspase-8 signaling complex [18]. The intrinsic pathway is controlled by the Bcl-2 family of proteins. First, the Bax/Bcl-2 ratio increases, and this increase is followed by the release of cytochrome c, leading to the activation of caspase-9 and caspase-3 [19]. The MAPK pathway is well known for its regulation of cell survival and apoptosis. The MAPK family is composed of three major kinases: ERK, p38, and JNK [20]. ERK is preferentially activated by growth factors, leading to cell growth and survival. p38 and JNK are preferentially activated by cytokine stimulation and oxidative stress, resulting in cell differentiation and apoptosis [21,22]. It is unclear whether the intrinsic/extrinsic pathway or the MAPK pathway is activated in PNAP-mediated apoptosis. In this study, we assessed the cytotoxicity of a series of PNAPs against MCF-7 cells and elucidated the mechanism underlying PNAP-induced cytotoxicity.

## Materials and Methods

### Chemistry

**Fig 1:**
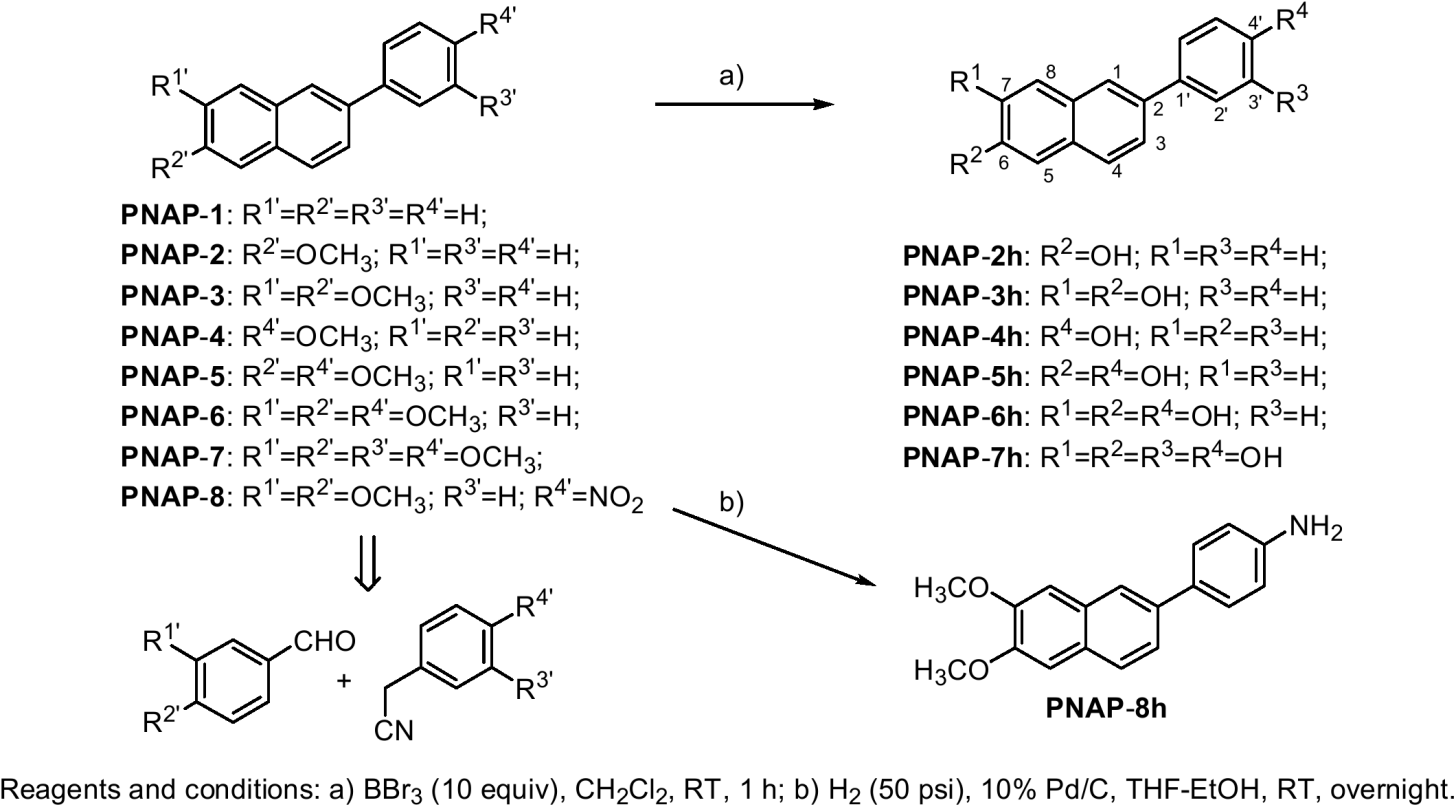
Synthetic approaches for the production of PNAP-1–PNAP-8 and PNAP-2h–PNAP-8h. The eight 2-phenylnaphthalenes (**PNAP**-**1**–**PNAP**-**8**) were easily obtained from phenylacetonitriles and benzaldehydes through six steps. Subsequently, the six hydroxy-PNAPs (**PNAP**-**2h**−**PNAP**-**7h**) were obtained by demethylation of the corresponding methoxy-PNAPs (**PNAP**-**2**−**PNAP**-**7**). Unfortunately, attempts at the demethylation of **PNAP**-**8** under the same conditions led to complex mixtures. Another hydrophilic amino-PNAP (**PNAP**-**8h**) was produced by the reduction of nitro-PNAP (**PNAP**-**8**).

The approaches used to synthesize the fifteen PNAP derivatives are shown in Fig 1. 2-Phenylnaphthalene (**PNAP**-**1**) and seven methoxy-PNAPs (**PNAP**-**2**−**PNAP**-**8**) were synthesized from commercially available phenylacetonitriles and benzaldehydes according to previously reported methods [23]. Six corresponding hydroxy-PNAPs (**PNAP**-**2h**−**PNAP**-**7h**) were obtained by the demethylation of **PNAP**-**2**−**PNAP**-**7** in excellent yields (91% to 100%). One amino-PNAP (**PNAP**-**8h**) was obtained by the hydrogenation of **PNAP**-**8** at a yield of 86%. The ^1^H and ^13^C NMR spectra of **PNAP**-**2h**−**PNAP**-**8h** are available in the Supporting Information (Figures A−G in S1 File). All of the PNAPs were dissolved in DMSO to a concentration of 50 mM to prepare stock solutions, which were stored at -20°C.

#### General Procedure for the Preparation of hydroxy-PNAPs

BBr_3_ in CH_2_Cl_2_ at a concentration of 1 M (5 mL, 5 mmol) was slowly added to a solution of methoxy-PNAPs (**PNAP**-**2**−**PNAP**-**7**, 0.5 mmol) in CH_2_Cl_2_ (20 mL) at 0°C. The mixture was allowed to warm to room temperature and was stirred for 1 h. The resulting solution was poured into H_2_O (50 mL). The H_2_O layer was extracted with EtOAc (3 × 50 mL), and the combined extracts were washed with H_2_O (3 × 50 mL), dried with anhydrous MgSO_4_, and filtered. The filtrate was concentrated, and the residue was purified by column chromatography over silica gel and eluted with EtOAc to obtain hydroxy-PNAPs (**PNAP**-**2h**−**PNAP**-**7h**). The full spectral data of these compounds are described as follows.

#### 6-Hydroxy-2-phenylnaphthalene (PNAP-2h)

Yield 91%; white solid, mp 176–177°C (lit. [24], mp 169−170°C). ^1^H NMR (500 MHz, CDCl_3_) *δ* 5.12 (1H, br s), 7.12 (1H, dd, *J* = 8.8, 2.4 Hz), 7.17 (1H, s), 7.36 (2H, t, *J* = 7.4 Hz), 7.47 (1H, t, *J* = 7.4 Hz), 7.69–7.71 (3H, m), 7.75 (1H, d, *J* = 8.8 Hz), 7.80 (1H, d, *J* = 8.8 Hz), 7.97 (1H, s); ^13^C NMR (125 MHz, CDCl_3_) *δ* 109.3, 118.2, 125.7, 126.3, 126.9, 127.1, 127.2 (2×C), 128.8 (2×C), 129.2, 130.2, 133.8, 136.4, 141.2, 153.5; IR (KBr) 3339, 3046, 1618, 1495, 1292, 1194 cm^-1^; ESIMS *m/z* (rel int) 219 (100, [M-H]^-^); HRESIMS *m/z* calcd for C_16_H_11_O: 219.08044; found: 219.08036 [M-H]^-^.

#### 6,7-Dihydroxy-2-phenylnaphthalene (PNAP-3h)

Yield 98%; white solid, mp 205–207°C. ^1^H NMR (500 MHz, DMSO-*d*_6_) *δ* 7.12 (1H, s), 7.20 (1H, s), 7.32 (1H, t, *J* = 7.5 Hz), 7.45 (2H, t, *J* = 7.5 Hz), 7.50 (1H, d, *J* = 8.4 Hz), 7.65 (1H, d, *J* = 8.4 Hz), 7.72 (2H, d, *J* = 7.5 Hz), 7.86 (1H, s), 9.57 (2H, br s); ^13^C NMR (125 MHz, DMSO-*d*_6_) *δ* 109.5, 110.2, 112.2, 123.4, 126.4, 126.8 (2×C), 127.0, 128.3, 129.0 (2×C), 129.3, 134.7, 140.9, 147.3, 147.4; IR (KBr) 3495, 3395, 1628, 1528, 1119 cm^-1^; ESIMS *m/z* (rel int) 235 (100, [M-H]^-^); HRESIMS *m/z* calcd for C_16_H_11_O_2_: 235.0754; found: 235.0755 [M-H]^-^.

#### 2-(4'-hydroxyphenyl)naphthalene (PNAP-4h)

Yield 100%; white solid, mp 166–167°C (lit. [25], mp 167−169°C). ^1^H NMR (500 MHz, CDCl_3_) *δ* 4.89 (1H, br s), 6.95 (2H, d, *J* = 8.6 Hz), 7.44–7.50 (2H, m), 7.61 (2H, d, *J* = 8.6 Hz), 7.70 (1H, dd, *J* = 8.5, 1.7 Hz), 7.84–7.90 (3H, m), 7.97 (1H, s); ^13^C NMR (125 MHz, CDCl_3_) *δ* 115.7 (2×C), 125.0, 125.4, 125.7, 126.2, 127.6, 128.0, 128.3, 128.7 (2×C), 132.3, 133.7, 133.9, 138.1, 155.1; IR (KBr) 3564, 3055, 1603, 1512, 1180 cm^-1^; ESIMS *m/z* (rel int) 219 (41, [M-H]^-^); HRESIMS *m/z* calcd for C_16_H_11_O: 219.08044; found: 219.08043 [M-H]^-^.

#### 6-hydroxy-2-(4'-hydroxyphenyl)naphthalene (PNAP-5h)

Yield 93%; white solid, mp 127–128°C (lit. [26], mp 258−262°C). ^1^H NMR (500 MHz, DMSO-*d*_6_) *δ* 6.86 (2H, d, *J* = 8.4 Hz), 7.01 (1H, dd, *J* = 8.8, 2.0 Hz), 7.10 (1H, s), 7.56 (2H, d, *J* = 8.4 Hz), 7.63 (1H, dd, *J* = 8.6, 1.6 Hz), 7.70 (1H, d, *J* = 8.6 Hz), 7.78 (1H, d, *J* = 8.8 Hz), 7.94 (1H, s), 9.51 (1H, br s), 9.70 (1H, br s); ^13^C NMR (125 MHz, DMSO-*d*_6_) *δ* 108.6, 115.9 (2×C), 119.1, 124.1, 125.2, 126.7, 127.8 (2×C), 128.3, 129.7, 131.2, 133.5, 134.6, 155.3, 157.0; IR (KBr) 3183, 3030, 1603, 1512, 1265, 1204 cm^-1^; ESIMS *m/z* (rel int) 235 (100, [M-H]^-^); HRESIMS *m/z* calcd for C_16_H_11_O_2_: 235.0754; found: 235.0751 [M-H]^-^.

#### 6,7-Dihydroxy-2-(4'-hydroxyphenyl)naphthalene (PNAP-6h)

Yield 98%; white solid, mp 280–282°C. ^1^H NMR (500 MHz, DMSO-*d*_6_) *δ* 6.84 (2H, d, *J* = 8.1 Hz), 7.08 (1H, s), 7.13 (1H, s), 7.42 (1H, d, *J* = 8.4 Hz), 7.53 (2H, d, *J* = 8.1 Hz), 7.58 (1H, d, *J* = 8.4 Hz), 7.73 (1H, s), 9.46 (3H, br s); ^13^C NMR (125 MHz, DMSO-*d*_6_) *δ* 109.5, 109.9, 115.8 (2×C), 122.0, 122.2, 126.2, 127.7, 127.8 (2×C), 129.3, 131.6, 134.8, 146.8, 147.3, 156.8; IR (KBr) 3495, 3341, 1597, 1520, 1119 cm^-1^; ESIMS *m/z* (rel int) 251 (100, [M-H]^-^); HRESIMS *m/z* calcd for C_16_H_11_O_3_: 251.07027; found: 251.07032 [M-H]^-^.

#### 6,7-Dihydroxy-2-(3',4'-dihydroxyphenyl)naphthalene (PNAP-7h)

Yield 100%; white solid, mp 230°C (decomp.). ^1^H NMR (500 MHz, DMSO-*d*_6_) *δ* 6.80 (1H, d, *J* = 8.2 Hz), 6.98 (1H, dd, *J* = 8.2, 2.0 Hz), 7.08 (1H, s), 7.09 (1H, d, *J* = 2.0 Hz), 7.13 (1H, s), 7.37 (1H, d, *J* = 8.4 Hz), 7.57 (1H, d, *J* = 8.4 Hz), 7.68 (1H, s), 8.94 (2H, br s), 9.46 (2H, br s); ^13^C NMR (125 MHz, DMSO-*d*_6_) *δ* 109.5, 110.0, 114.1, 116.2, 117.7, 122.0, 122.2, 126.2, 127.7, 129.3, 132.3, 135.1, 144.9, 145.7, 146.8, 147.3; IR (KBr) 3372, 3329, 1602, 1234, 1111 cm^-1^; ESIMS *m/z* (rel int) 267 (100, [M-H]^-^); HRESIMS *m/z* calcd for C_16_H_11_O_4_: 267.0652; found: 267.0647 [M-H]^-^.

#### 2-(4'-Aminophenyl)-6,7-dimethoxynaphthalene (PNAP-8h)

**PNAP**-**8** (77 mg, 0.25 mmol) was subjected to hydrogenation (50 psi) using 10% Pd/C as the catalyst in tetrahydrofuran (THF, 10 mL) and EtOH (1 mL) at room temperature for 12 h. The reaction mixture was filtered, and the filtrate was concentrated. The residue was purified by column chromatography over silica gel and eluted with EtOAc to yield **PNAP**-**8h**. Yield 86%; pale yellow solid, mp 190–191°C. ^1^H NMR (500 MHz, CDCl_3_) *δ* 3.74 (2H, br s), 4.01 (6H, s), 6.79 (2H, d, *J* = 8.1 Hz), 7.12 (1H, s), 7.15 (1H, s), 7.52 (2H, d, *J* = 8.1 Hz), 7.56 (1H, d, *J* = 8.4 Hz), 7.71 (1H, d, *J* = 8.4 Hz), 7.82 (1H, s); ^13^C NMR (125 MHz, CDCl_3_) *δ* 55.9 (2×C), 106.1, 106.5, 115.5 (2×C), 123.2, 123.6, 126.7, 127.8, 128.1 (2×C), 129.6, 131.8, 137.0, 145.6, 149.2, 149.7; IR (KBr) 3460, 3439, 3018, 2995, 1624, 1504, 1244, 1130 cm^-1^; ESIMS *m/z* (rel int) 280 (100, [M+H]^+^); HRESIMS *m/z* calcd for C_18_H_17_NO_2_: 251.07027; found: 251.07032 [M+H]^+^.

### Cell culture

MCF-7 cells were obtained from the laboratory of Dr. Yang-Chang Wu (School of Pharmacy, China Medical University, Taiwan), and MCF-10A cells were obtained from the laboratory of Dr. Wei-Chien Huang (Graduate Institute of Cancer Biology, China Medical University, Taiwan). MCF-7 cells were maintained in DMEM/F12 medium containing 10% fetal bovine serum and 1% penicillin-streptomycin. MCF-10A cells were maintained in DMEM/F12 medium containing 1.05 mM CaCl_2_, 100 mg/ml cholera toxin, 5% horse serum, 10 μg/ml insulin, 500 ng/ml hydrocortisone and 1% penicillin-streptomycin. These cells were cultured in cell culture incubators, which were set to temperatures of 37°C and supplemented with 5% CO_2_.

### Cell viability assay

MCF-7 cells were plated at a density of 5×10^3^ cells per well in 96-well plates, incubated overnight and then treated with different concentrations (0, 0.5, 1, 2.5, 5, 10, 25, and 50 μM) of fifteen PNAPs (**PNAP**-**1**−**PNAP**-**8** and **PNAP**-**2h**−**PNAP**-**8h**). In addition, MCF-10A cells were plated at a density of 5×10^3^ cells per well in 96-well plates, incubated overnight and then treated with different concentrations (0, 1, 2.5, 5, 10, 25, 50, and 100 μM) of three PNAPs (**PNAP**-**3h**, -**6h**, and -**7h**). After 48 h of treatment, 10 μl of MTT solution (5 mg/ml in PBS) was added to each well, and the cells were incubated for an additional 4 h at 37°C. The medium was then completely removed, and 50 μl of DMSO was added to solubilize the MTT formazan crystals. The absorbance at a wavelength of 550 nm was then measured using a MQX200R microplate reader (BioTek, VT, USA).

### Cell cycle analysis

MCF-7 cells were seeded at a density of 3×10^5^ cells per well in six-well plates, incubated overnight and then treated with **PNAP**-**1**, -**3h**, and -**6h** at three different concentrations (5, 10, and 25 μM). After 48 h of treatment, the cells were trypsinized, washed once with PBS, and fixed in 70% ethanol for 1 h at -20°C. The fixed cells were washed with ice-cold PBS, suspended in 0.5 ml of PBS containing 0.2 mg/ml RNase and 0.1% Triton X-100 for 30 min at room temperature, and stained with 20 μg/ml propidium iodide. The stained cells were analyzed using a FACScan flow cytometer (Becton Dickinson, CA, USA). The fluorescence emitted from the propidium iodide-DNA complex was estimated from a minimum of 10,000 cells per sample and analyzed using the Cell Quest Alias software (BD Biosciences, USA).

### Western blot analysis

MCF-7 cells were seeded at a density of 3×10^5^ cells per well in six-well plates, incubated overnight and then treated with **PNAP**-**1**, -**3h**, and -**6h** at three different concentrations (5, 10, and 25 μM) for 48 h. The cells were washed with PBS and lysed in ice-cold RIPA buffer for 30 min. The supernatant was collected and centrifuged at 15,000 rpm and 4°C for 30 min. The protein concentration was measured through a Bio-Rad assay using a MQX200R microplate reader (BioTek, VT, USA). The cell lysates were separated by 10% SDS-PAGE and electrophoretically transferred onto polyvinylidene difluoride (PVDF) membranes (Millipore, Bedford, MA, USA). After several washes, the membranes were blocked with 5% skim milk in TBST (Tris-buffered saline containing 0.1% Tween-20) for 1 h at room temperature and incubated overnight at 4°C with different primary antibodies against cyclin D1, CDK4, cyclin E, CDK2, p21, p27, cyclin B1, CDK1, cleaved caspase-7, -8, -9, PARP, Bcl-2, Bcl-xl, Bax, Bid, Fas, p-p38, p38, p-JNK, JNK, p-ERK, or ERK (1:1000 dilution). The membranes were then washed three times with TBST and probed with horseradish peroxidase (HRP)-conjugated secondary antibody (1:2000) for 1 h at room temperature. After three washes in TBST, the bound antibody was visualized using the ECL Western Blotting Reagent (PerkinElmer, Boston, MA, USA), and the chemiluminescence was detected using Fuji Medical X-ray film (Tokyo, Japan).

### Cell morphology studies

The cells were plated in 12-well plates at a density of 1×10^5^ cells/well, incubated overnight and then treated with **PNAP**-**1**, -**3h**, and -**6h** at three different concentrations (5, 10, and 25 μM) for 48 h. The cells were washed once with PBS, and a subset of cells was stained with Hoechst 33342 (10 μg/ml for 15 min). The morphological changes were then observed by light microscopy at 100× magnification and fluorescence microscopy at 200× magnification.

### Statistical analysis

All of the data are expressed as the means ± SEM from three replicate experiments. The differences among the groups were compared by analysis of variance (ANOVA) and post-hoc Tukey Honestly Significant Difference (HSD) test using the SPSS 12.0 software for Windows (USA). P values less than 0.05 were considered statistically significant.

## Results and Discussion

### Effect of PNAPs on MCF-7 cell line viability and study of their structure-activity relationship

To investigate the effects of the 2-phenylnaphthalenes **PNAP**-**1**−**PNAP**-**8**, their demethylation derivatives **PNAP**-**2h**−**PNAP**-**7h**, and amino-PNAP (**PNAP**-**8h**) on cell survival, MCF-7 cells were treated with different doses of each compound (0, 0.5, 1, 2.5, 5, 10, 25, and 50 μM) for 48 h. The rates of viability were determined via the MTT assay, and the results are shown in Table 1. First, **PNAP**-**1** showed no significant activity in MCF-7 cells, and **PNAP**-**2**−**PNAP**-**8**, which contain methoxy groups, caused precipitation in the cell media at concentrations higher than 5 μM (Table 1). Therefore, the more hydrophilic PNAP derivatives **PNAP**-**2h**−**PNAP**-**8h** were designed and synthesized. Indeed, no precipitation was observed for **PNAP**-**2h**−**PNAP**-**8h**, most likely due to the formation of intermolecular hydrogen bonds between water and the hydroxyl (or amino) groups on the 2-phenylnaphthalenes. Three of the derivatives (**PNAP**-**3h**, -**6h**, and -**7h**) exhibited significant dose-dependent toxicity against MCF-7 cells, with IC_50_ values of 17.9, 4.8, and 31.8 μM, respectively (Table 1), whereas the IC_50_ values obtained against MCF-10A cells were 71.0, 50.9, and 55.1 μM, respectively. The results indicate that **PNAP**-**3h** and -**6h** possess enhanced and selective cytotoxicity against MCF-7 cells and show low toxicity toward MCF-10A cells.

**Table 1:** Effect of 2-Phenylnaphthalenes PNAP-1−PNAP-8, Their Demethylation Derivatives PNAP-2h−PNAP-7h, and Amino PNAP-8h on the Viability of MCF-7 Cells.

Compound	Viability (% of control)^a^							
	0 μM	0.5 μM	1.0 μM	2.5 μM	5.0 μM	10.0 μM	25.0 μM	50.0 μM
**PNAP-1**	100.0	97.3 ± 2.6	94.6 ± 1.2	97.0 ± 1.7	91.9 ± 1.7	96.1 ± 2.2	92.6 ± 2.3	86.1 ± 0.4
**PNAP-2**	100.0	97.5 ± 3.4	97.4 ± 2.4	100.7 ± 3.0	101.6 ± 1.9	83.1 ± 4.9	56.5 ± 2.1	59.0 ± 2.0
**PNAP-3**	100.0	98.6 ± 0.7	97.1 ± 0.7	96.7 ± 1.3	95.8 ± 1.4	88.9 ± 0.5	52.9 ± 0.6	51.8 ± 0.9
**PNAP-4**	100.0	96.2 ± 3.8	97.0 ± 2.2	100.4 ± 2.8	102.7 ± 2.2	96.8 ± 2.8	61.7 ± 2.4	56.0 ± 1.5
**PNAP-5**	100.0	94.7 ± 1.6	94.8 ± 1.9	96.5 ± 2.2	93.2 ± 0.8	89.5 ± 1.0	77.3 ± 1.8	60.4 ± 2.1
**PNAP-6**	100.0	95.2 ± 3.4	94.7 ± 2.2	76.7 ± 4.8	50.2 ± 0.7	49.2 ± 1.1	48.3 ± 2.2	48.9 ± 1.5
**PNAP-7**	100.0	90.4 ± 2.5	89.8 ± 1.1	88.1 ± 1.8	68.0 ± 3.6	50.7 ± 1.6	48.3 ± 0.7	47.6 ± 0.7
**PNAP-8**	100.0	89.5 ± 1.8	88.5 ± 3.8	78.6 ± 1.9	66.5 ± 0.5	52.1 ± 1.0	47.0 ± 1.2	48.0 ± 0.8
**PNAP-2h**	100.0	104.9 ± 0.9	102.5 ± 0.9	102.3 ± 1.5	102.1 ± 0.8	97.2 ± 1.9	82.7 ± 4.4	60.0 ± 3.2**
**PNAP-3h**	100.0	98.6 ± 1.5	97.4 ± 4.2	94.8 ± 2.0	86.8 ± 2.0	77.6 ± 1.1**	32.9 ± 1.3**	18.7 ± 2.5**
**PNAP-4h**	100.0	102.8 ± 1.9	100.3 ± 4.5	102.0 ± 8.2	103.7 ± 3.6	102.3 ± 2.2	95.5 ± 3.8	78.0 ± 4.9
**PNAP-5h**	100.0	100.5 ± 1.2	101.1 ± 2.6	100.6 ± 1.7	97.7 ± 0.8	77.3 ± 5.8*	70.2 ± 2.7*	56.2 ± 2.2*
**PNAP-6h**	100.0	101.8 ± 1.8	96.3 ± 1.1	72.3 ± 3.1*	48.4 ± 3.9**	40.9 ± 3.9**	18.2 ± 3.5**	12.4 ± 2.0**
**PNAP-7h**	100.0	97.1 ± 1.7	98.6 ± 3.2	97.5 ± 1.0	99.6 ± 0.6	85.0 ± 3.6	55.3 ± 0.9**	40.3 ± 2.4**
**PNAP-8h**	100.0	99.9 ± 0.8	98.9 ± 2.4	101.1 ± 1.9	89.3 ± 5.4	93.2 ± 2.1	85.2 ± 5.8	75.3 ± 0.9*

The MCF-7 cell line was treated with **PNAP**-**1**−**PNAP**-**8**, and **PNAP**-**2h**−**PNAP**-**8h** at eight concentrations and then incubated at 37°C for 48 h. The cell viability was examined via the MTT assay.

^a^ The number of viable cells remaining after PNAP treatment is expressed as a percentage of the vehicle control, which was arbitrarily assigned a value of 100.0%. The results are presented as the means ± SEM from three independent assays.

*P<0.05 and **P<0.01 compared with the control.

The above-described results revealed that **PNAP**-**5h**, which has hydroxyl groups at the C-6 position of the naphthalene ring and at the C-4**'** position of the phenyl ring, exhibited better cytotoxicity than **PNAP**-**1**, -**2h**, and -**4h** in our tests. This phenomenon shows that the introduction of hydroxyl groups at the C-6 position of the naphthalene ring and the C-4**'** position of the phenyl ring enhances cytotoxicity. Intriguingly, the two 2-phenylnaphthalenes **PNAP**-**3h** and -**6h**, which contain a hydroxyl group at the C-7 position of the naphthalene ring, exhibited better cytotoxic activities against the MCF-7 cell line (compare **PNAP**-**3h** to -**2h** and compare **PNAP**-**6h** to -**5h**). These results suggest that cytotoxicity is markedly promoted by the presence of a hydroxyl group at the C-7 position of the naphthalene ring. Moreover, it should be noted that **PNAP**-**6h** exhibited the best activity against the MCF-7 cell line (IC_50_ = 4.8 μM). This result also indicated that the introduction of a hydroxyl group at the C-4**'** position of the phenyl ring would enhance the activities against the MCF-7 cell line. In contrast, the presence of a hydroxyl group at the C-3**'** position of the phenyl ring in **PNAP**-**7h** may markedly diminish cytotoxicity (compare **PNAP**-**6h** and -**7h**). In addition, the comparison of the cytotoxic activities of **PNAP**-**6h** and **PNAP**-**8h** suggests that the presence of a hydroxyl group, which serves as a H-bond donor, on the naphthalene ring yields better activities than the presence of a methoxy group, which serves as a H-bond acceptor. Based on the cell viability and SAR results, we further investigated the potential reasons for the decrease in cell viability induced by **PNAP**-**3h** and -**6h** and elucidated the underlying mechanism.

### Effect of PNAPs on the morphological characteristics of MCF-7 cells

**Fig 2:**
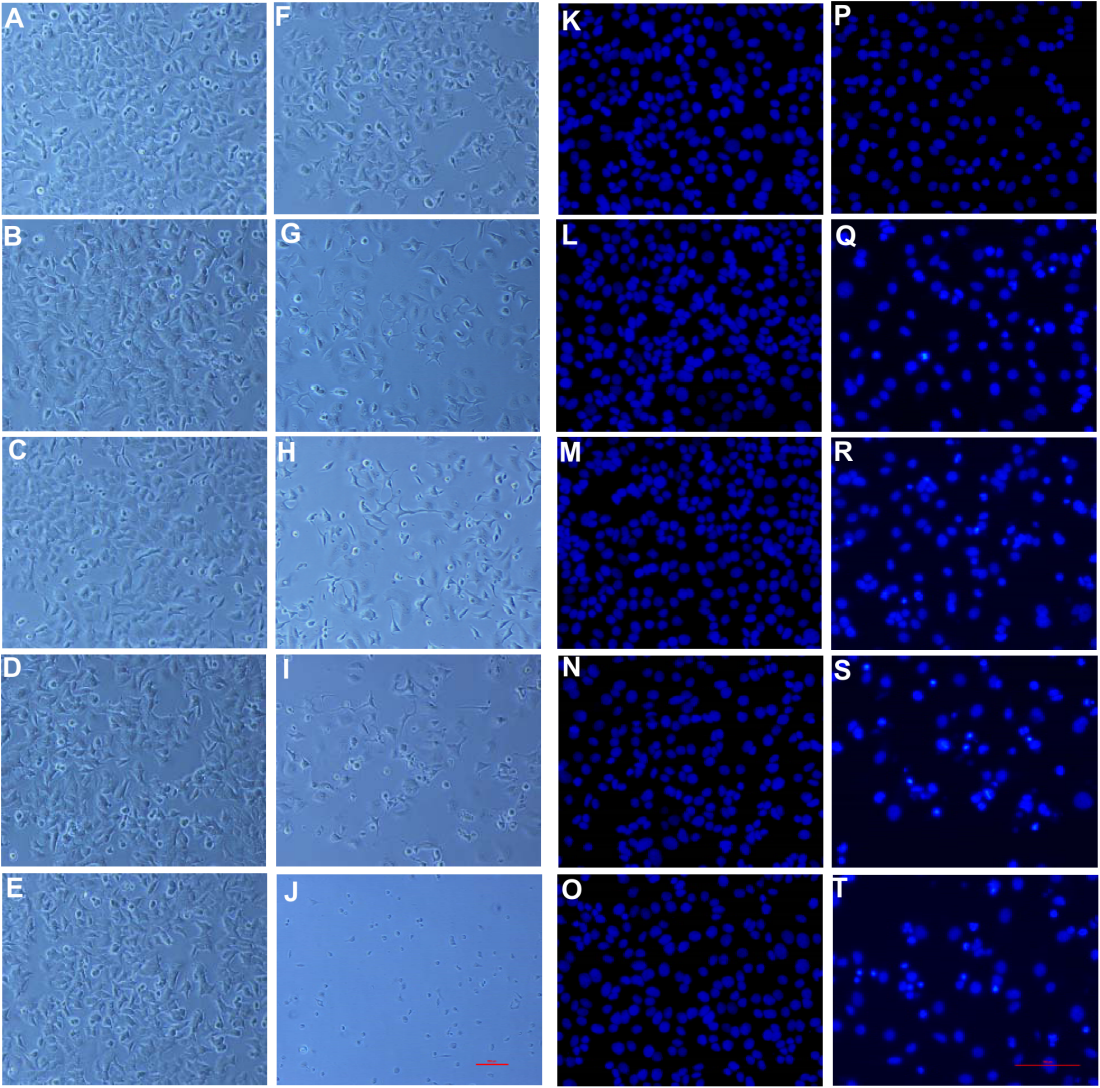
Effect of PNAP-1, -3h, and -6h on MCF-7 morphology. MCF-7 cells were treated with **PNAP**-**1**, -**3h**, and -**6h** at three concentrations (5, 10, and 25 μM) for 48 h, and then, (A–J) the morphology was observed by light microscopy (100×). The cells were stained with Hoechst 33342, and then, (K–T) the morphology was observed by fluorescence microscopy (200×). (A/K) control; (B/L) 5 μM **PNAP**-**1**; (C/M) 10 μM **PNAP**-**1**; (D/N) 25 μM **PNAP**-**1**; (E/O) 5 μM **PNAP**-**3h**; (F/P) 10 μM **PNAP**-**3h**; (G/Q) 25 μM **PNAP**-**3h**; (H/R) 5 μM **PNAP**-**6h**; (I/S) 10 μM **PNAP**-**6h**; and (J/T) 25 μM **PNAP**-**6h**.

First, the effect of **PNAP**-**1**, -**3h** and -**6h** on cellular morphology was observed by phase-contrast microscopy. MCF-7 cells treated with either a vehicle control (0.05% DMSO) or 5 to 25 μM **PNAP**-**1** showed dense cell populations under a phase-contrast microscope (Fig 2A–2D). The MCF-7 cells were regular in shape and size, with eccentric nuclei and a relatively small amount of cytoplasm. The cell density and structure of cells treated for 48 h with 5 to 25 μM **PNAP**-**3h** and -**6h** showed obvious changes (Fig 2E–2J). The cells became rounded and shrunken and lost contact with adjacent cells. In addition, the apoptotic cells no longer adhered to the substrate, causing them to detach from the culture plates and float in the culture medium. Thus, **PNAP**-**3h** and -**6h** led to a decrease in the viable MCF-7 cell numbers in a concentration-dependent manner, as shown in Table 1.

We also observed the nuclear morphology of MCF-7 cells subjected to Hoechst 33342 fluorescent staining after treatment for 48 h with three concentrations (5, 10, and 25 μM) of **PNAP**-**1**, -**3h**, and -**6h** (Fig 2L–2T). The nuclei of the vehicle control cells displayed an intact oval shape and were exhibited a weak blue fluorescence (Fig 2K). However, the cells treated with **PNAP**-**3h** (25 μM) and **PNAP**-**6h** (5, 10, and 25 μM) exhibited typical apoptotic features, such as nuclear shrinkage, nuclear fragmentation, and nuclear hypercondensation (Fig 2Q–2T). Based on these microscopic observations, we propose that the observed cytotoxicity may be partially mediated through **PNAP**-**3h-** and -**6h**-induced apoptosis.

### Effect of PNAPs on MCF-7 cell cycle phase distribution

**Fig 3:**
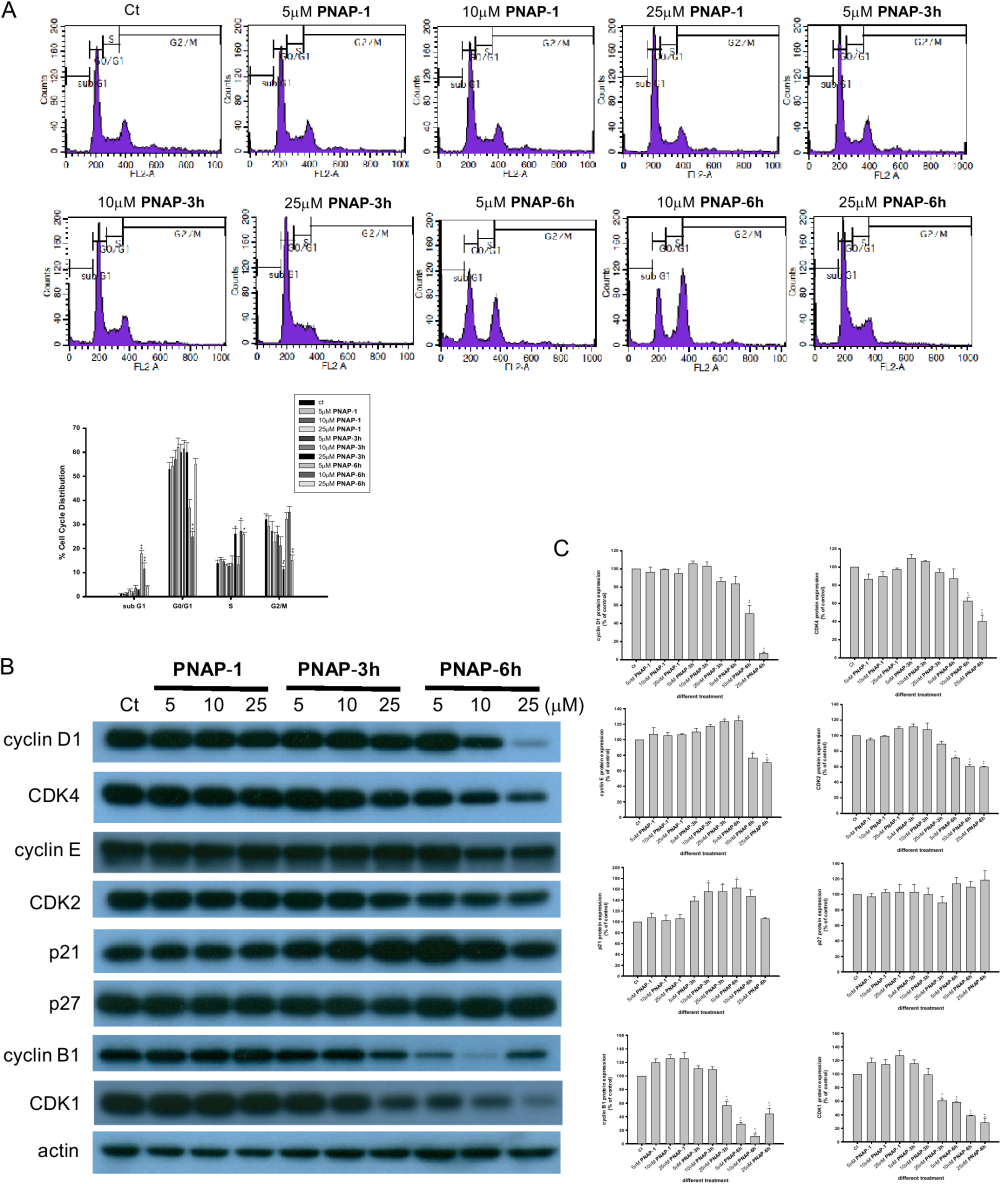
Effect of PNAP-1, -3h, and -6h on MCF-7 cell cycle progression. MCF-7 cells were treated with **PNAP**-**1**, -**3h**, and -**6h** at three concentrations (5, 10, and 25 μM) for 48 h, and then, (A) the cells were fixed and stained with propidium iodide to analyze the DNA content by flow cytometry. (B, C) Cell cycle-associated proteins (cyclin D1, CDK4, cyclin E, CDK2, p21, p27, cyclin B1, and CDK1) were detected and analyzed by western blot. The expression was quantified with the computerized Gel-Pro Analyzer image analysis system. The data are expressed as the means ± SEM from three independent assays. *P<0.05 and **P<0.01 are significantly different from the control.

Because cell proliferation is regulated by the cell cycle, the effect of **PNAP**-**1**, -**3h** and -**6h** on cell cycle progression in the MCF-7 cell line was examined by flow cytometry and western blot analyses. The results of the flow cytometric analysis indicated that treatment with **PNAP**-**6h** for 48 h increased the sub-G_1_ population (Fig 3A). The sub-G_1_ peak, which consisted of cells with reduced DNA content, represented the presence of apoptotic cells [27,28]. In addition, the **PNAP**-**6h**-induced cell arrest at the G_2_/M phase obtained at lower concentrations (5 and 10 μM) was accompanied by a decrease in the percentage of cells found at the Go/G1 phase. **PNAP**-**5h** and -**6h** caused S phase arrest at a higher concentration (25 μM), led to a lower number of replicating cells and suppressed cell proliferation. However, the **PNAP**-**1**-treated cells showed no significant change in the cell cycle distribution compared with the vehicle control cells.

Based on these observations of the cell cycle distribution of MCF-7 cells, we analyzed the changes in the abundance of cell cycle regulatory proteins. Cyclin-CDK complexes are the principal regulators of cell cycle progression [7], and the activity of cyclin/CDK complexes are negatively regulated by CDK inhibitors, such as p21 and p27 [29]. **PNAP**-**3h** (at 25 μM) and **PNAP**-**6h** led to cell arrest at the S phase, most likely due to increased levels of p21 and p27 and reduced levels of cyclin D1, CDK4, cyclin E, and CDK2, as determined through the western blot analysis (Fig 3B and 3C). In addition, **PNAP**-**6h** induced an inhibition of CDK1 kinase activity and a decrease in cyclin B1 expression, most likely leading to G_2_/M arrest. Therefore, we propose that the inhibition of cell viability by **PNAP-3h** and -**6h** may be partially mediated through cell cycle arrest and apoptosis.

### PNAPs activate the caspase pathway and increase the expression of relative apoptotic proteins

**Fig 4:**
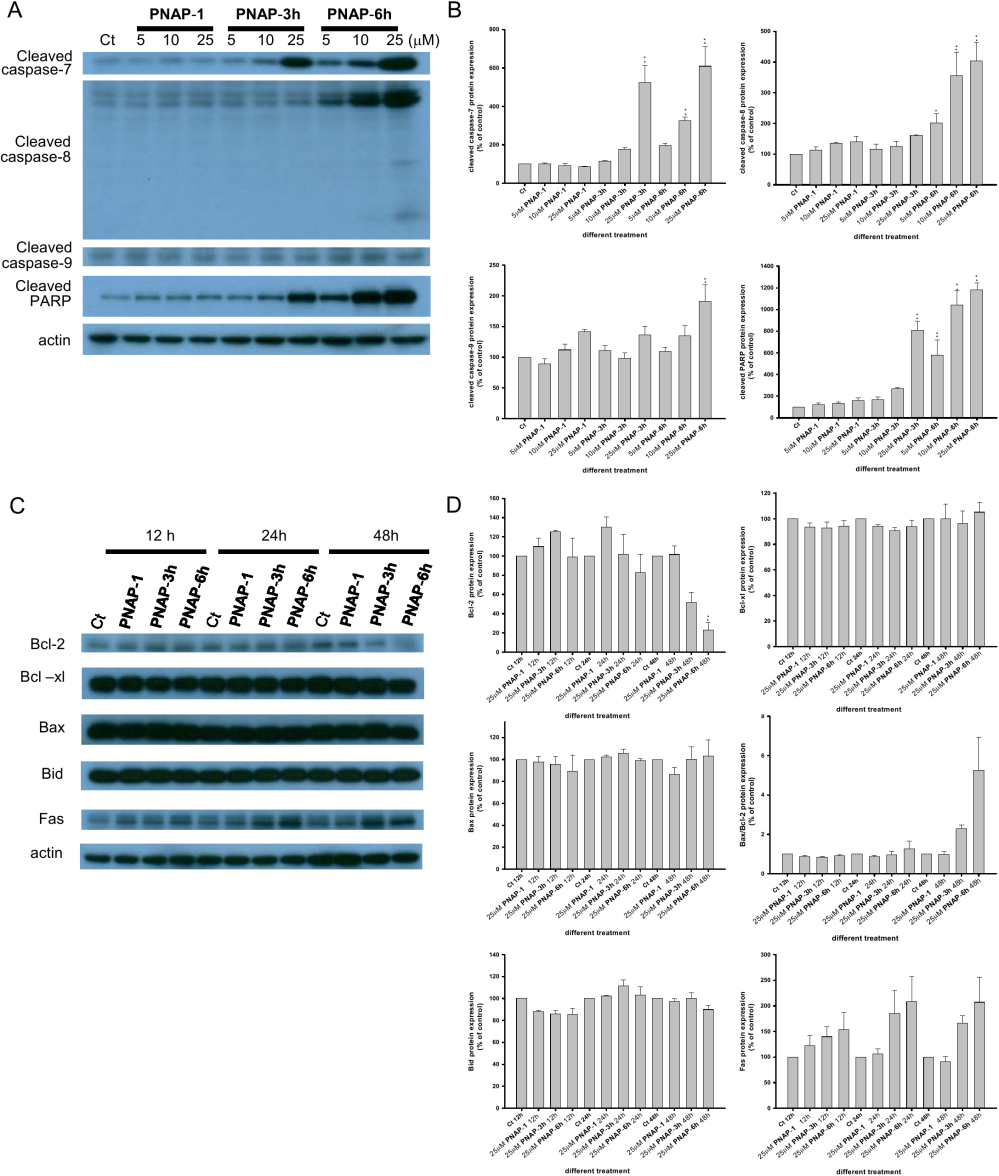
Effect of PNAP-1, -3h, and -6h on the expression of apoptosis-related proteins. (A) MCF-7 cells were treated with **PNAP**-**1**, -**3h**, and -**6h** at three concentrations (5, 10, and 25 μM) for 48 h, and the expression of cleaved caspase-7, -8, -9 and PARP was determined by western blotting. (C) MCF-7 cells were treated with 25 μM **PNAP**-**1**, -**3h**, and -**6h** at three concentrations (5, 10, and 25 μM) for 48 h, and the Bcl-2, Bcl-xl, Bax, Bax/Bcl-2, Bid, and Fas levels were then detected by western blot. (B, D) The expression was quantified using a computerized Gel-Pro Analyzer image analysis system. The data are expressed as the means ± SEM from three independent assays. *P<0.05 and **P<0.01 are significantly different from the control.

**Fig 5:**
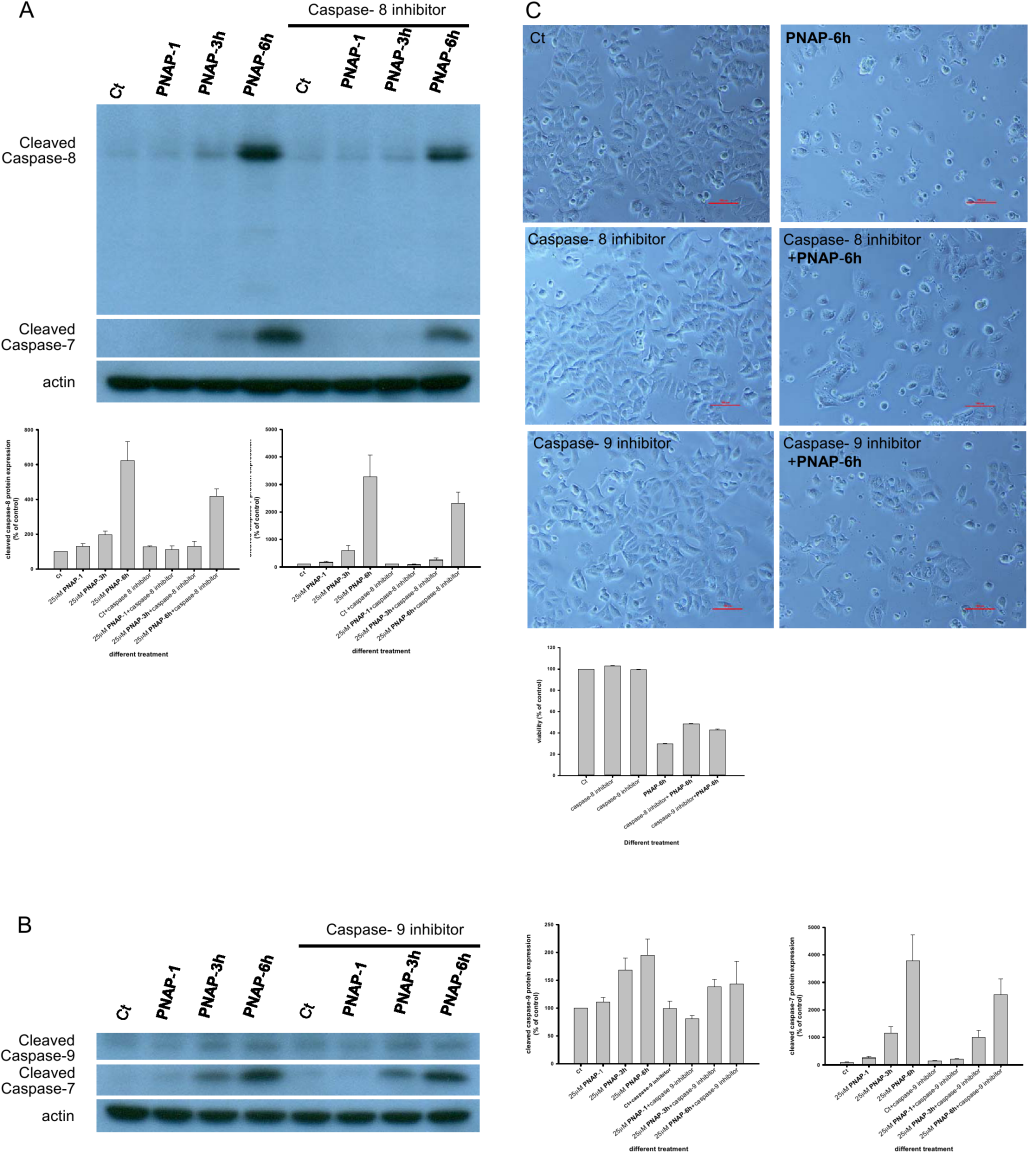
Effect of PNAP-6h on the expression of caspase-7, -8, and -9 proteins. MCF-7 cells were incubated in the presence or absence of (A) the caspase-8 inhibitor z-IETD-FMK or (B) the caspase-9 inhibitor z-LEHD-FMK for 1.5 h before the addition of **PNAP**-**6h**. Western blot analyses were conducted with antibodies to caspase-7, -8, and -9, and actin. (C) MCF-7 cells were treated with **PNAP**-**6h** in the presence or absence of caspase-8 inhibitor, or caspase-9 inhibitor, and the morphology was then observed by light microscopy.

Based on the above results, we found that PNAPs can prevent proliferation, induce apoptosis, and decrease cell viability in the MCF-7 cell line. It is well known that numerous anticancer drugs mediate apoptosis by activating caspases [16]. To demonstrate that the PNAP-induced apoptosis in MCF-7 cells occurs via caspase activation, we investigated the expression of key regulatory proteins associated with the caspase pathways in MCF-7 cells upon exposure to 5 to 25 μM **PNAP**-**1**, -**3h**, and -**6h**. Despite the lack of caspase-3 in MCF-7 cells, caspase-7, an apoptosis executioner, may function as a replacement [30]. Caspase-7 is capable of cleaving specific tetrapeptide substrates (e.g., PARP), and PARP cleavage is a major hallmark of apoptosis [31]. The exposure of MCF-7 cells to three concentrations (5, 10, and 25 μM) of **PNAP**-**3h** and -**6h** for 48 h significantly increased the levels of cleaved caspase-7 and PARP and increased the activity of cleaved caspase-7 and PARP in a dose-dependent manner (Fig 4A). Furthermore, apoptosis could be stimulated via the extrinsic and intrinsic pathways. Caspase-8 is generally considered an apoptosis activator in the extrinsic pathway, and caspase-9 plays an important role in the intrinsic pathway [32]. These data show that the expression of cleaved caspase-8 and caspase-9 in MCF-7 cells increased after treatment with **PNAP**-**3h** and -**6h** in a dose-dependent manner. Furthermore, cleaved caspase-8 and caspase-9 were partially inhibited by the caspase-8-specific inhibitor Z-IETD-FMK and the caspase-9-specific inhibitor Z-LEHD-FMK. Moreover, the two inhibitors also partially inhibited caspase-7 cleavage in PNAP derivative-induced apoptosis (Fig 5A and 5B) and partially reversed the **PNAP**-**6**-induced decrease in cell viability, as observed by microscopy and the MTT assay (Fig 5C). These results suggest that the caspase-7-, caspase-8-, and caspase-9-mediated pathways may be partially involved in the regulation of PNAP-induced apoptosis.

The activation of caspase-8 is the first step in the cascade of apoptotic events induced by Fas stimulation [33], and the activation of caspase-9 is regulated by Bcl-2 family members [34–36]. However, the treatment of MCF-7 cells with 25 μM PNAP derivatives exerted a modest effect on the expression of Bcl-xl, Bax, and Bid proteins at three time points (12, 24, and 48 h) (Fig 4C and 4D). In contrast, the expression of Bcl-2 protein was significantly decreased and the ratio of Bax/Bcl-2 was increased in a time-dependent manner (12, 24, and 48 h). We also found that **PNAP**-**3h** and -**6h** significantly induced the expression of Fas protein. Taken together, these findings suggest that the treatment of MCF-7 cells with **PNAP**-**3h** and -**6h** leads to an increase in the Bax/Bcl-2 ratio and Fas levels, possibly inducing the activation of caspase-8 and -9.

### PNAPs induce p38 and ERK activation

**Fig 6:**
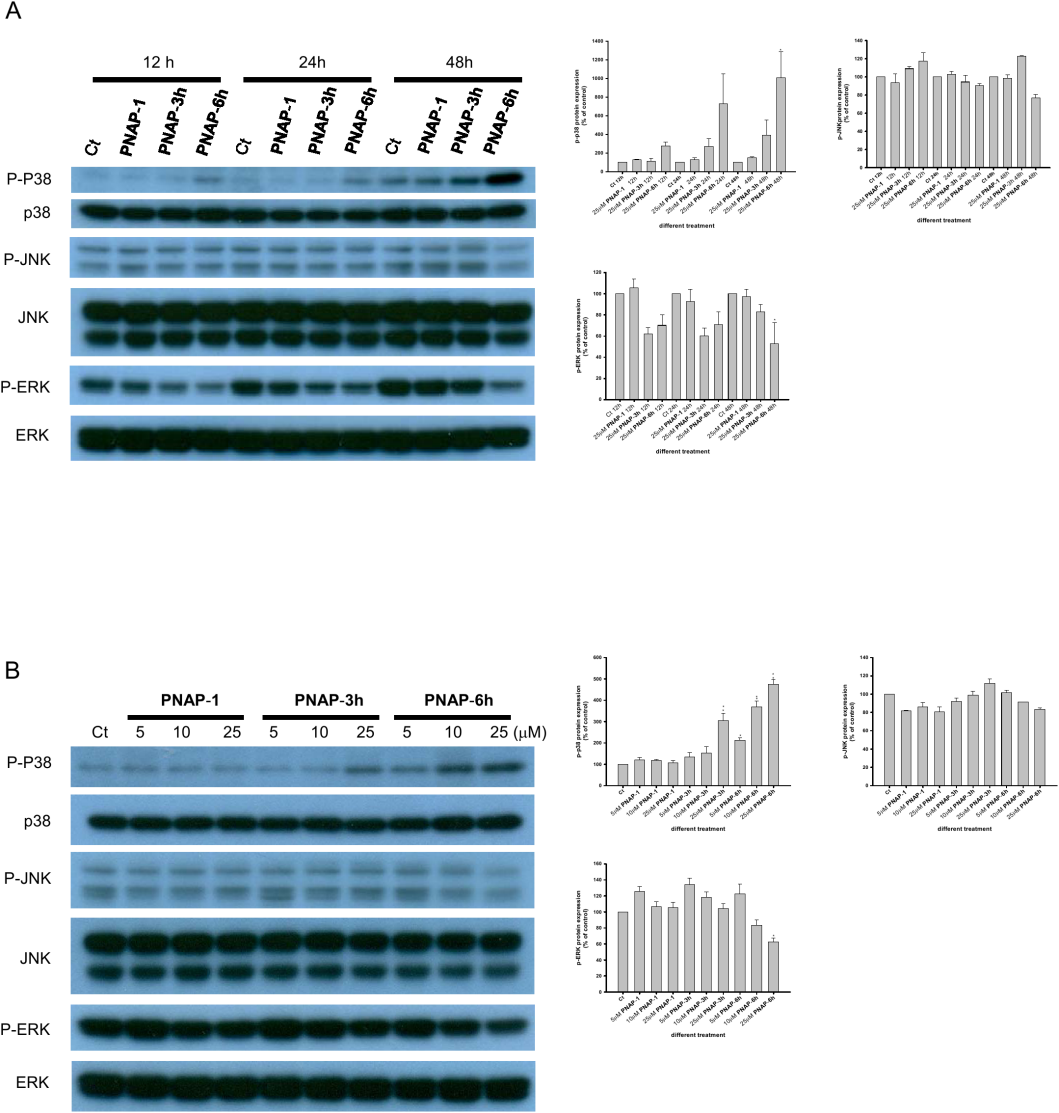
Expression of MAPKs in PNAP-induced apoptosis. (A) MCF-7 cells were treated with 25 μM **PNAP**-**1**, -**3h**, and -**6h** at three time points (12, 24, and 48 h). (B) MCF-7 cells were treated with **PNAP**-**1**, -**3h**, and -**6h** at three concentrations (5, 10, and 25 μM) for 48 h. ERK, p38, and JNK, as well as their respective phosphorylated forms, were then analyzed by western blot. The data are expressed as the means ± SEM from three independent assays. *P<0.05 and **P<0.01 are significantly different from the control.

The MAPK family plays a central role in the signaling pathways of cell proliferation, differentiation, and apoptosis [37]. Therefore, the involvement of the MAPK pathway in the PNAP derivative-mediated apoptosis of MCF-7 cells was examined. The expression levels of MAPK (ERK, p38, and JNK) proteins during PNAP derivative-induced apoptosis at three time points (12, 24, and 48 h) and at different PNAP concentrations (5, 10, and 25 μM) are shown in Fig 6A and 6B, respectively. p38 and JNK kinases are mainly activated by extracellular stress, resulting in cell differentiation and apoptosis [38]. In this study, we found that **PNAP**-**3h** and -**6h** treatment but not **PNAP**-**1** treatment significantly increased the p38 phosphorylation levels in MCF-7 cells in a time- and dose-dependent manner; however, the total amount of p38 protein did not change. This result shows an increase in the phosphorylation status of p38 and indicates that **PNAP**-**3h** and -**6h** increases p38 activity (Fig 6A and 6B). In contrast to the increase in p38 activity, no elevation in the phosphorylation of JNK or in the total JNK levels was observed after treatment with **PNAP**-**1**, -**3h**, and -**6h**. In addition, **PNAP**-**6h** decreased the expression of p-ERK in a dose-dependent manner (Fig 6B) because ERK is generally activated by mitogenic and proliferative stimuli and is involved in cellular proliferation and survival [39]. These results indicate that p38 and ERK are also associated with **PNAP**-**3h** and -**6h**-induced apoptosis of MCF-7 cells.

## Conclusion

**Fig 7:**
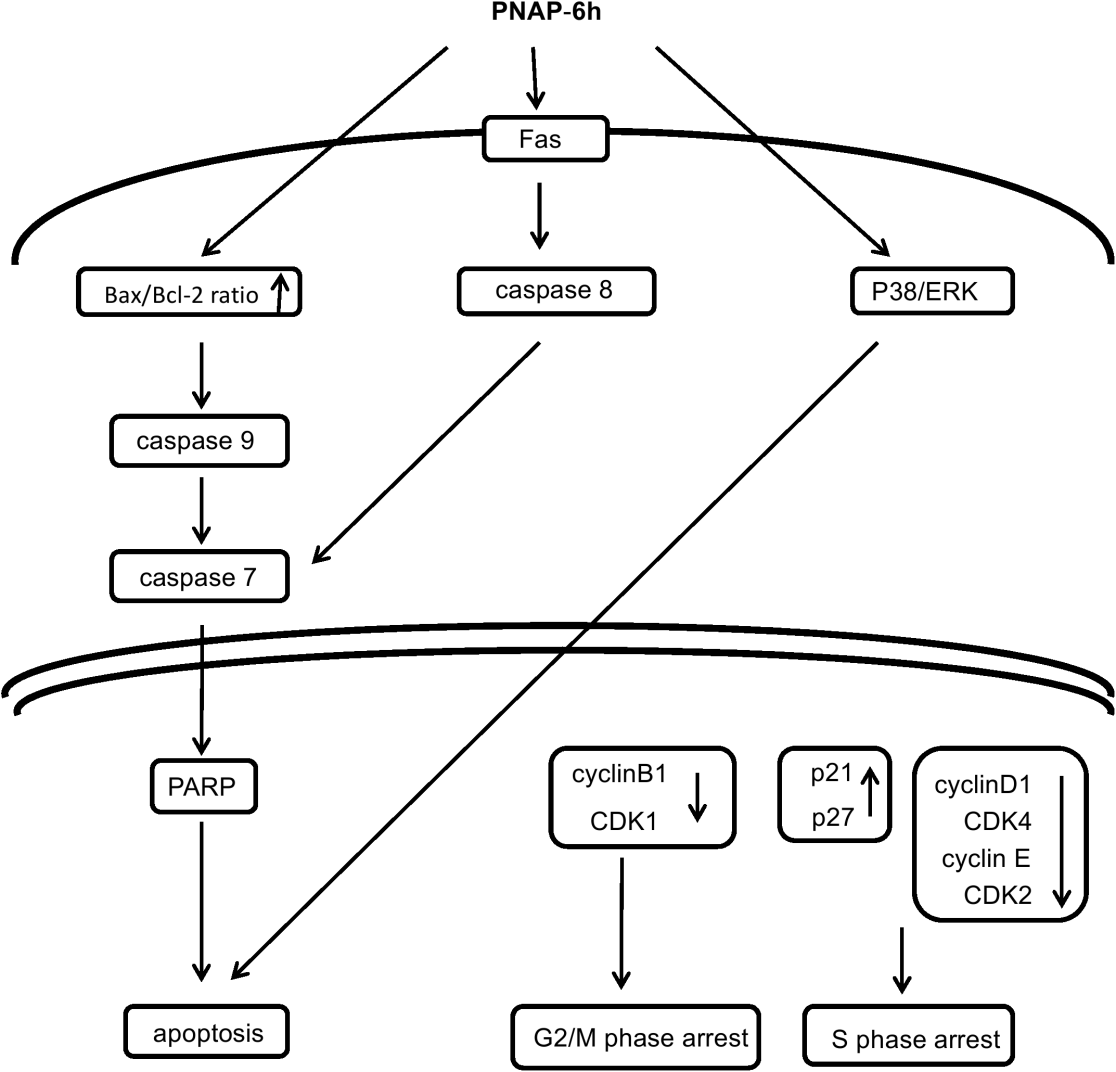
Proposed model of PNAP-6h-mediated MCF-7 cell cycle arrest and apoptosis. **PNAP**-**6h** causes cell cycle arrest at the S and G2/M phases. **PNAP**-**6h** modulates MCF-7 apoptosis via activation of the intrinsic/extrinsic pathways and the p38/ERK pathway.

In summary, a series of hydrophilic PNAP derivatives was designed and synthesized. **PNAP**-**6h**, a 6,7-dihydroxy-2-phenylnaphthalene with a *para*-hydroxyl group on the phenyl ring, exhibited the strongest cytotoxicity in our tests. In addition, our SAR studies revealed that 7-hydroxy-2-phenylnaphthalene was the active core. Moreover, **PNAP**-**6h** treatment resulted in changes in cellular morphology and induced cell cycle arrest and apoptosis in a dose-dependent manner. We proposed a model for **PNAP**-**6**-mediated MCF-7 cell cycle arrest and apoptosis, as shown in Fig 7. **PNAP**-**6h** induced S phase arrest via the promotion of p21 and p27 and the inhibition of cyclin D1, CDK4, cyclin E, and CDK2. In addition, G_2_/M phase arrest was mediated by reductions in the expression of cyclin B1 and CDK1. **PNAP**-**6h** induced apoptosis in MCF-7 cells not only via the intrinsic and extrinsic pathways but also through the p38 and ERK pathways.

## Supporting Information

S1 File The ^1^H and ^13^C NMR spectra of PNAP-2h−PNAP-8h. ^1^H and ^13^C NMR spectra of **PNAP-2h** (**Figure A**). ^1^H and ^13^C NMR spectra of **PNAP-3h** (**Figure B**). ^1^H and ^13^C NMR spectra of **PNAP-4h** (**Figure C**). ^1^H and ^13^C NMR spectra of **PNAP-5h** (**Figure D**). ^1^H and ^13^C NMR spectra of **PNAP-6h** (**Figure E**). ^1^H and ^13^C NMR spectra of **PNAP-7h** (**Figure F**). ^1^H and ^13^C NMR spectra of **PNAP-8h** (**Figure G**).
(PDF)
