# Supplementary material for: Structure-Activity Relationship of Synthetic 2-Phenylnaphthalenes with Hydroxyl Groups that Inhibit Proliferation and Induce Apoptosis of MCF-7 Cancer Cells
Source: PLoS One. 2015 Oct 22;10(10):e0141184. doi: 10.1371/journal.pone.0141184 (PMC4619615; doi:10.1371/journal.pone.0141184)
Supplement: S1 File — 1H and 13C NMR spectra of PNAP-2h (Figure A). 1H and 13C NMR spectra of PNAP-3h (Figure B). 1H and 13C NMR spectra of PNAP-4h (Figure C). 1H and 13C NMR spectra of PNAP-5h (Figure D). 1H and 13C NMR spectra of PNAP-6h (Figure E). 1H and 13C NMR spectra of PNAP-7h (Figure F). 1H and 13C NMR spectra of PNAP-8h (Figure G). (PDF) [file pone.0141184.s001.pdf]

^{1}H NMR (500MHz, $CDCl_3$)

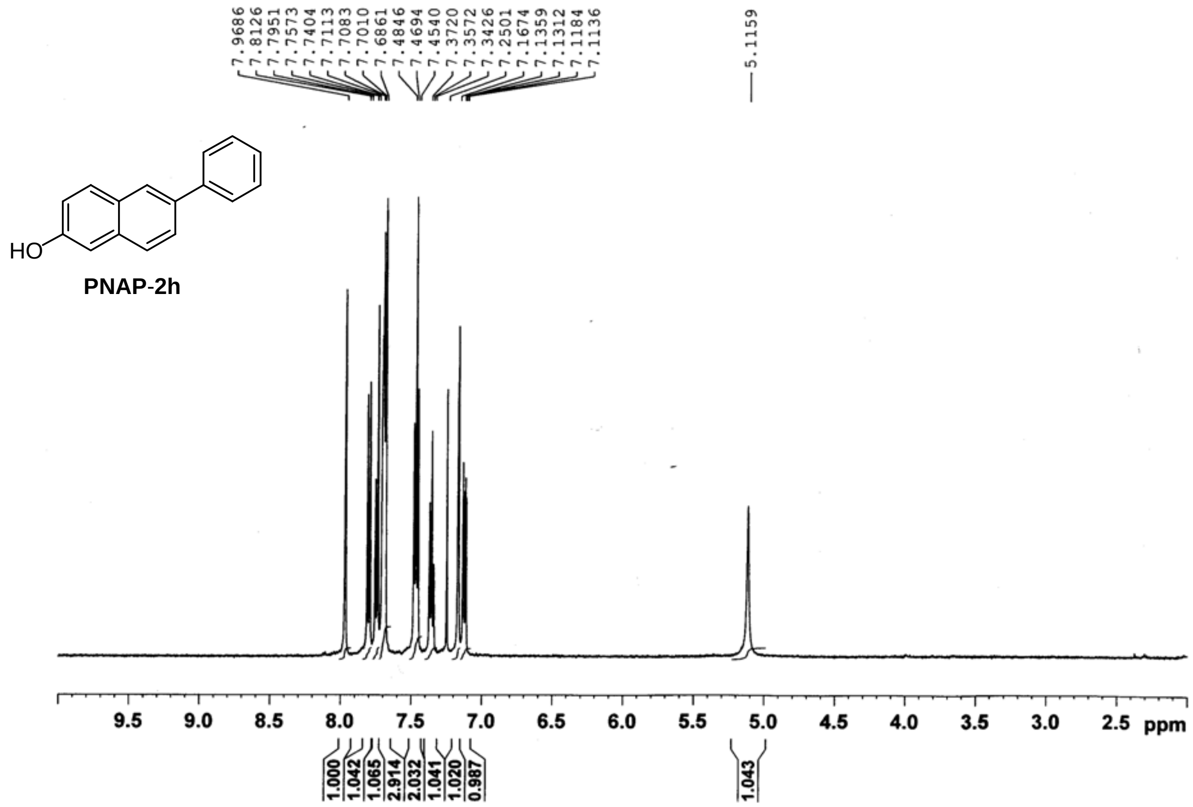

^{13}C NMR (125MHz, $CDCl_3$)

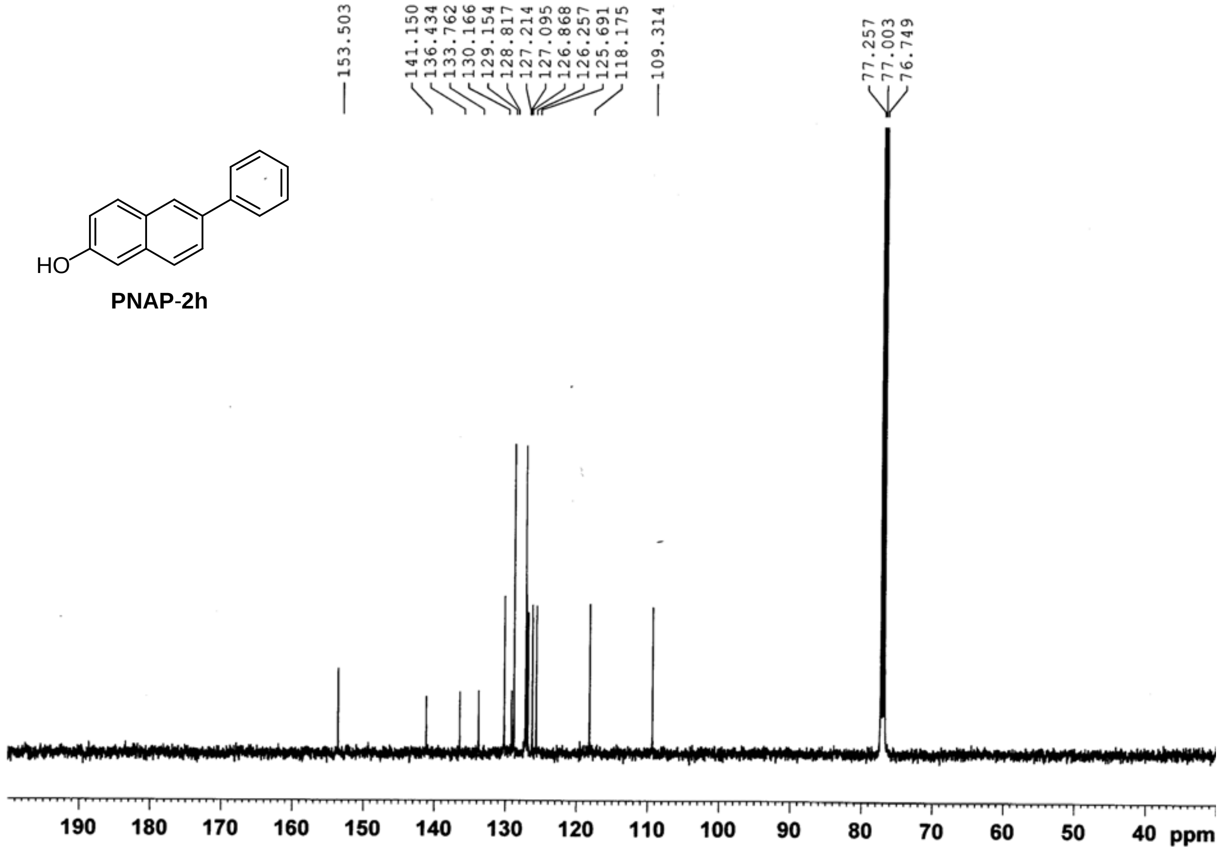

Figure A. ^{1}H and ^{13}C NMR spectra of PNAP-2h.

^{1}H NMR (500MHz, DMSO-d_6)

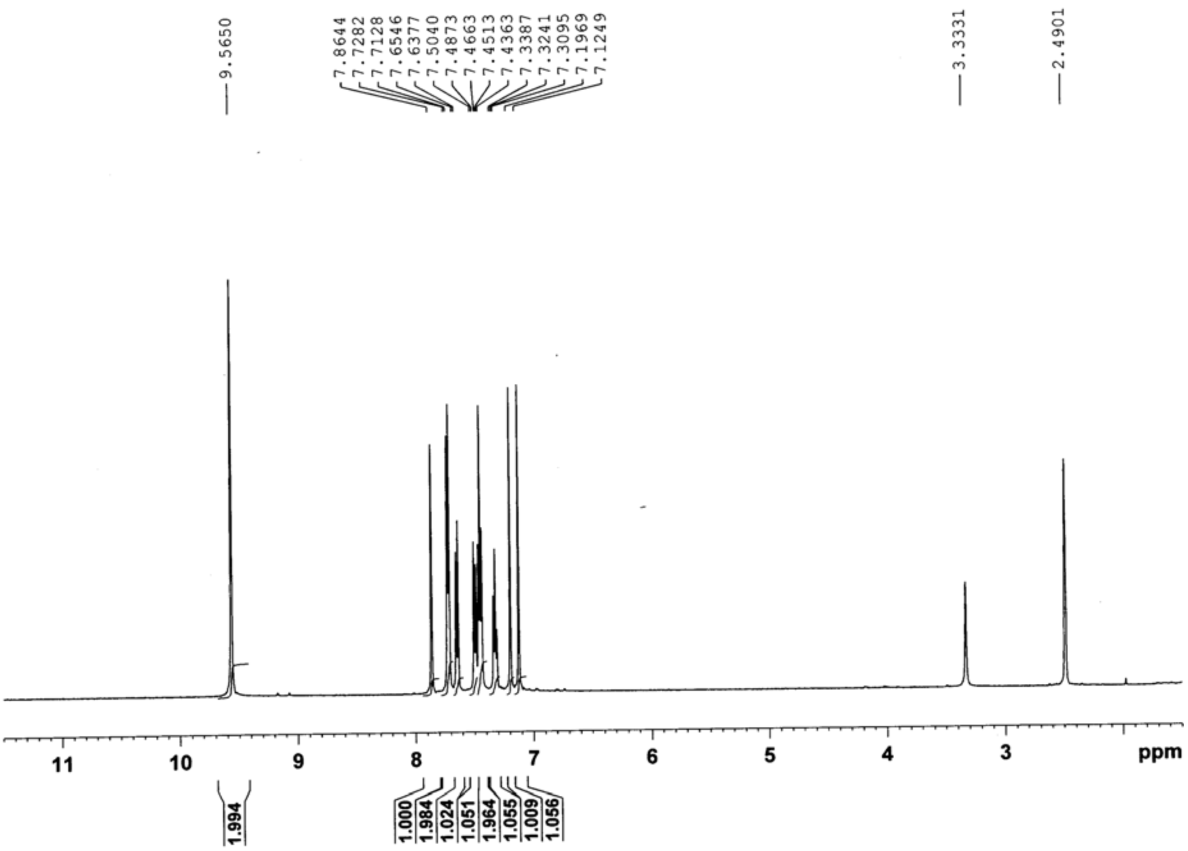

^{13}C NMR (125MHz, DMSO-d_6)

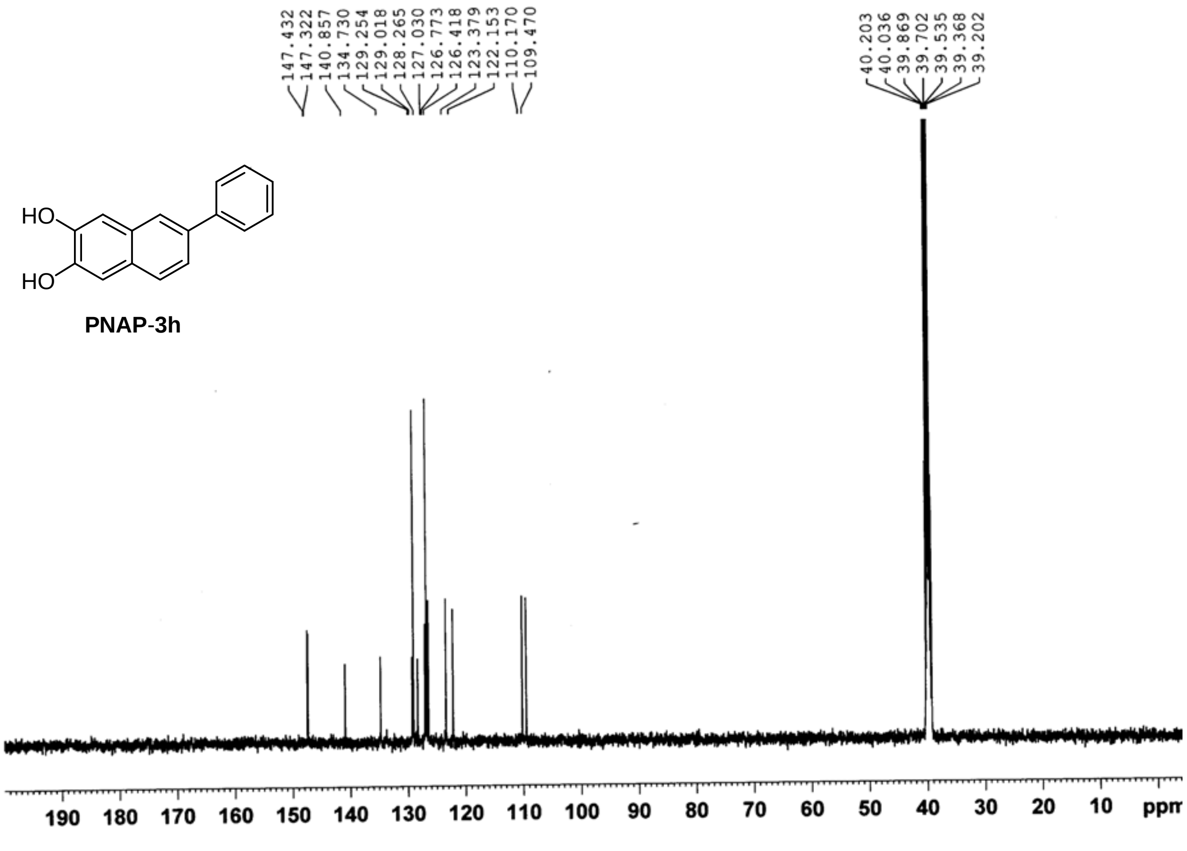

Figure B. ^{1}H and ^{13}C NMR spectra of PNAP-3h.

^{1}H NMR (500MHz, $CDCl_3$)

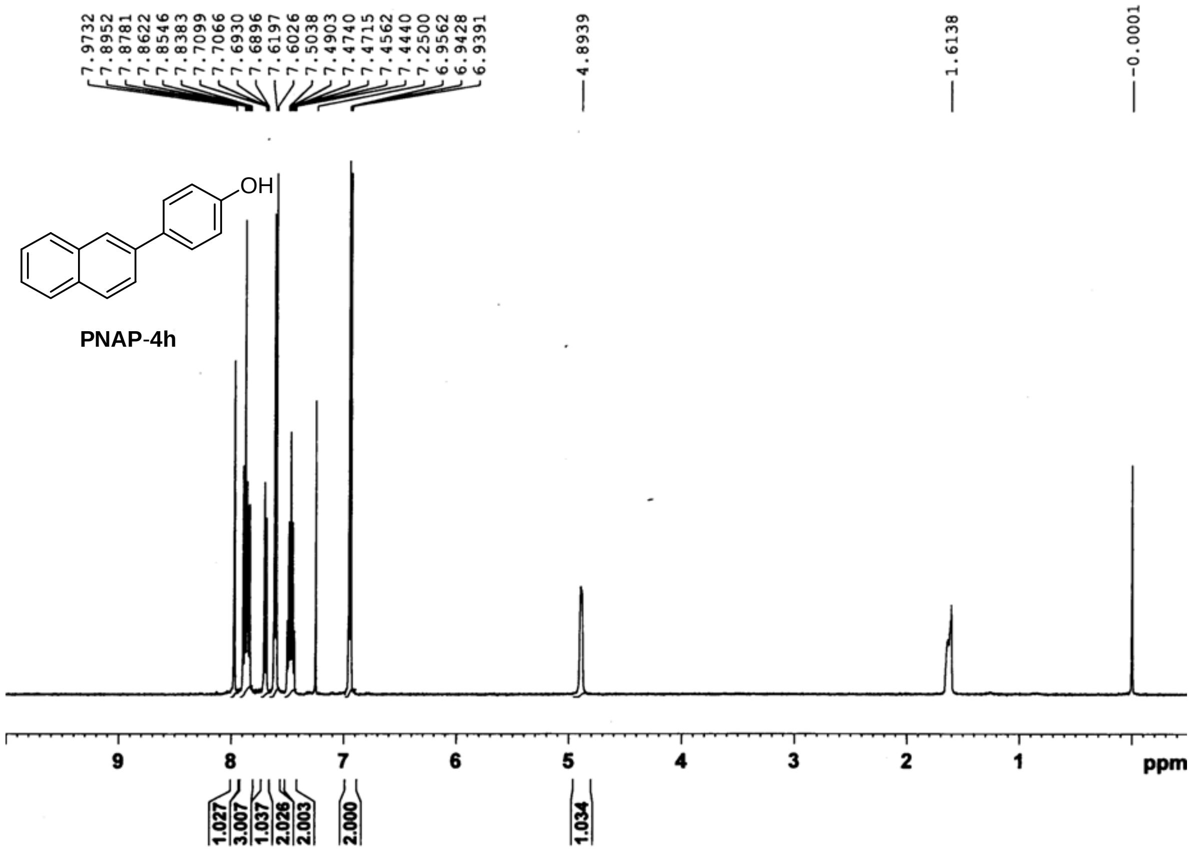

^{13}C NMR (125MHz, $CDCl_3$)

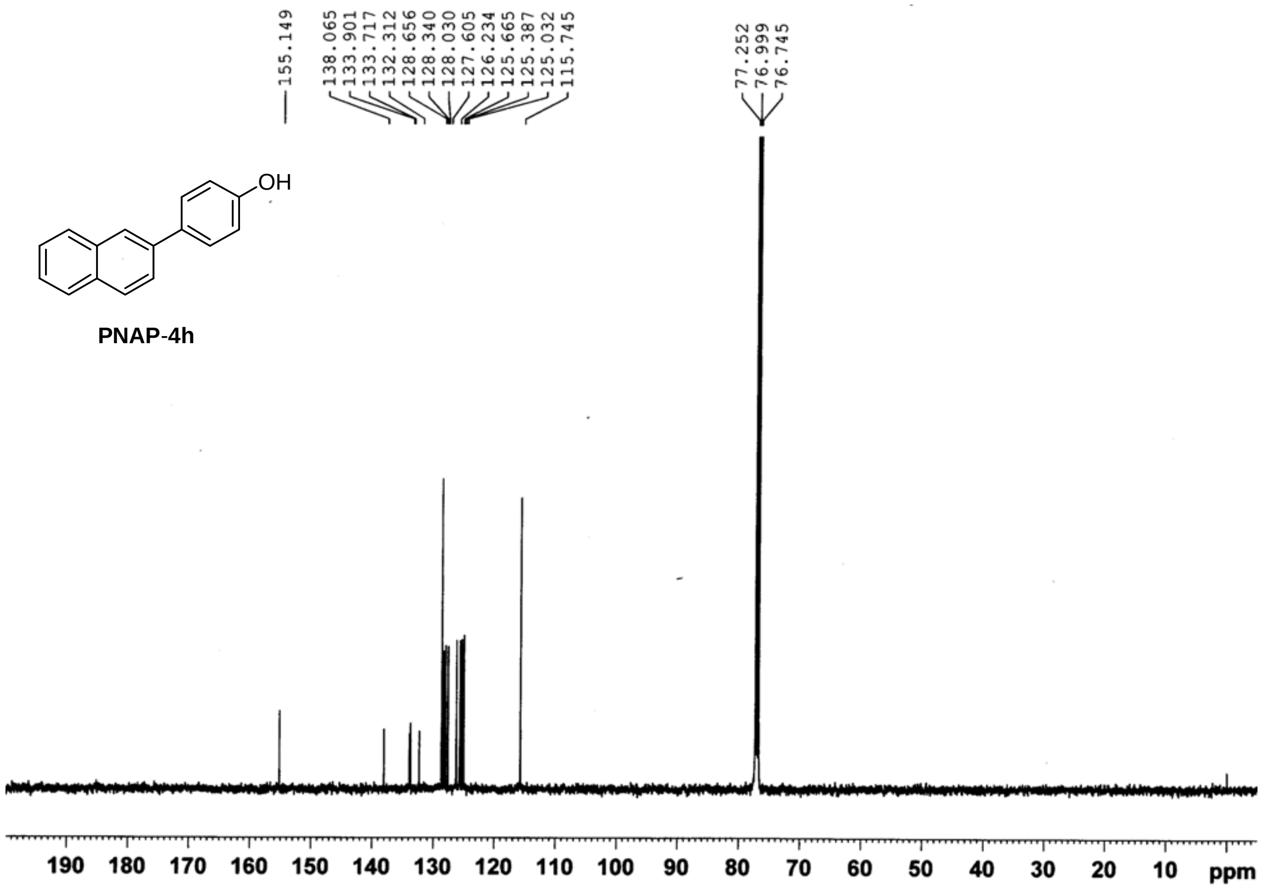

Figure C. ^{1}H and ^{13}C NMR spectra of PNAP-4h.

^{1}H NMR (500MHz, DMSO-d_6)

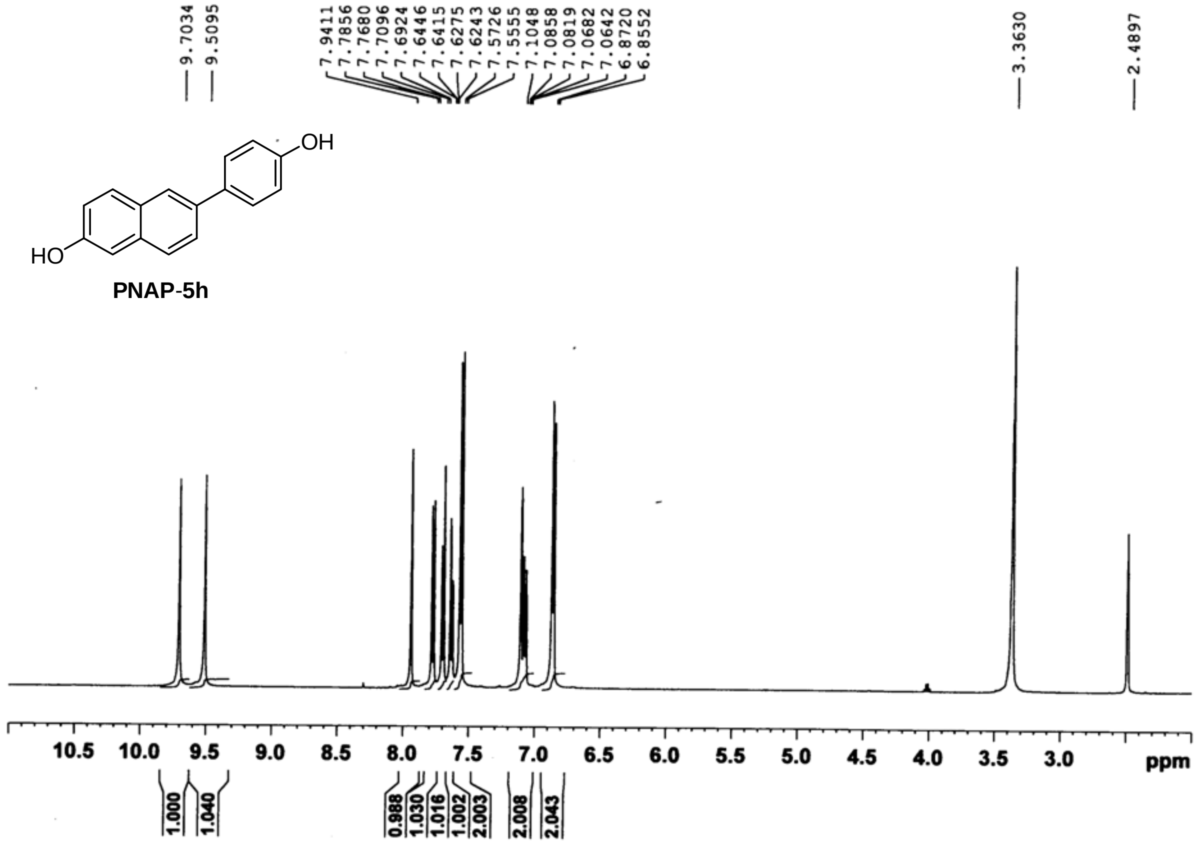

^{13}C NMR (125MHz, DMSO-d_6)

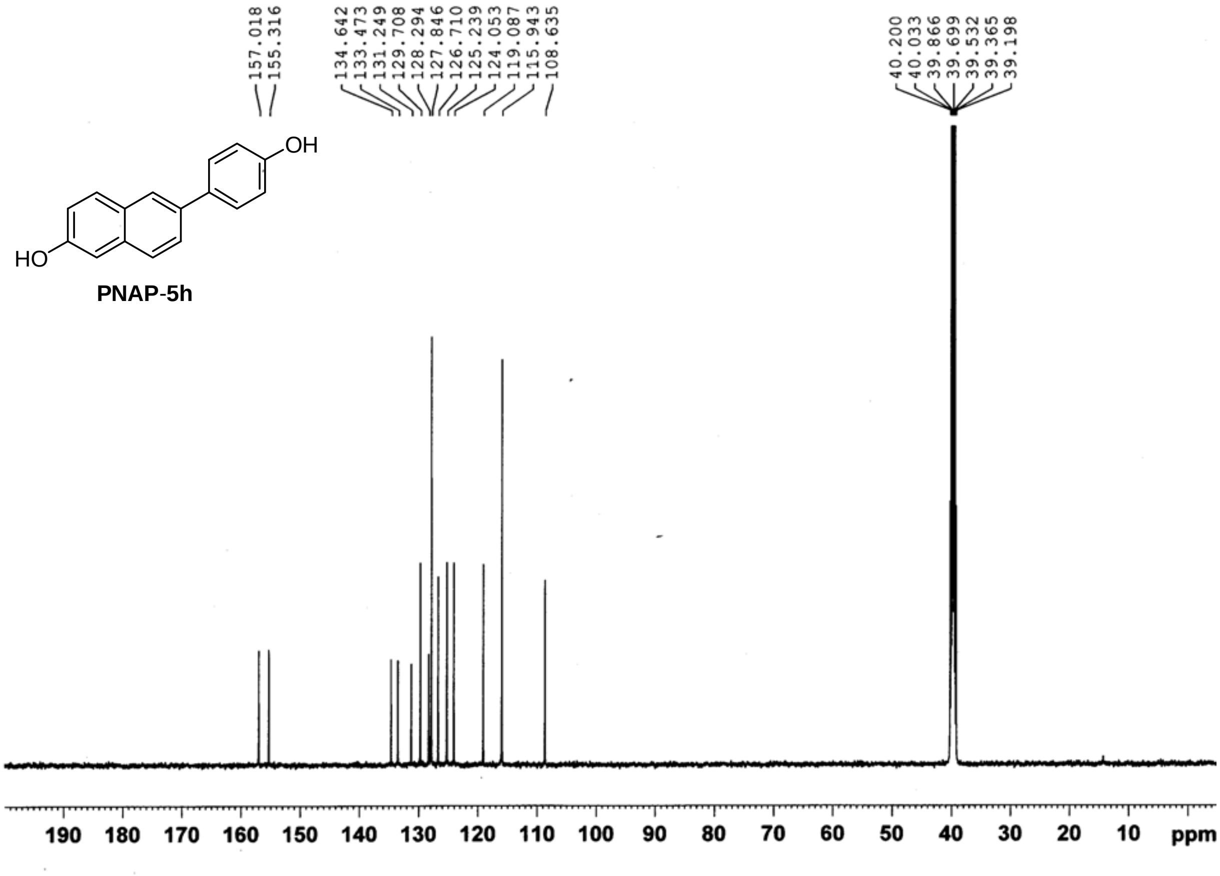

Figure D. ^{1}H and ^{13}C NMR spectra of PNAP-5h.

^{1}H NMR (500MHz, DMSO-d_6)

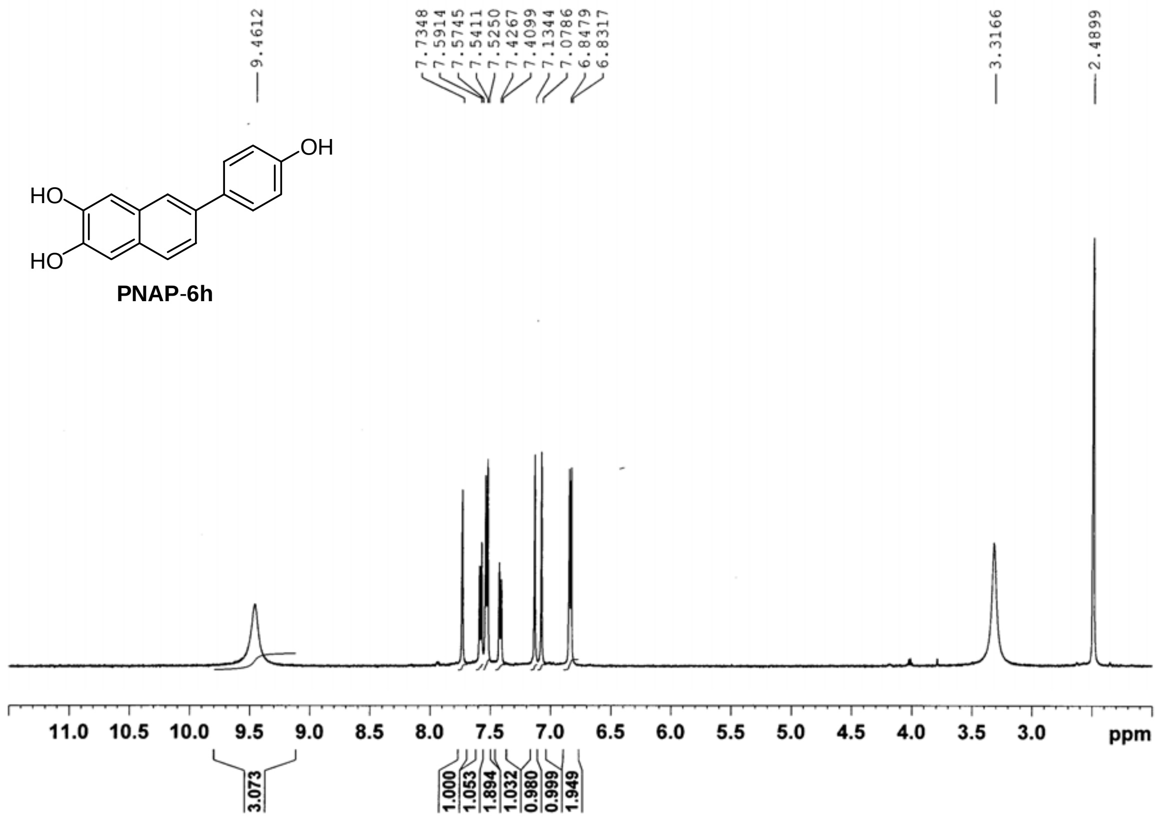

^{13}C NMR (125MHz, DMSO-d_6)

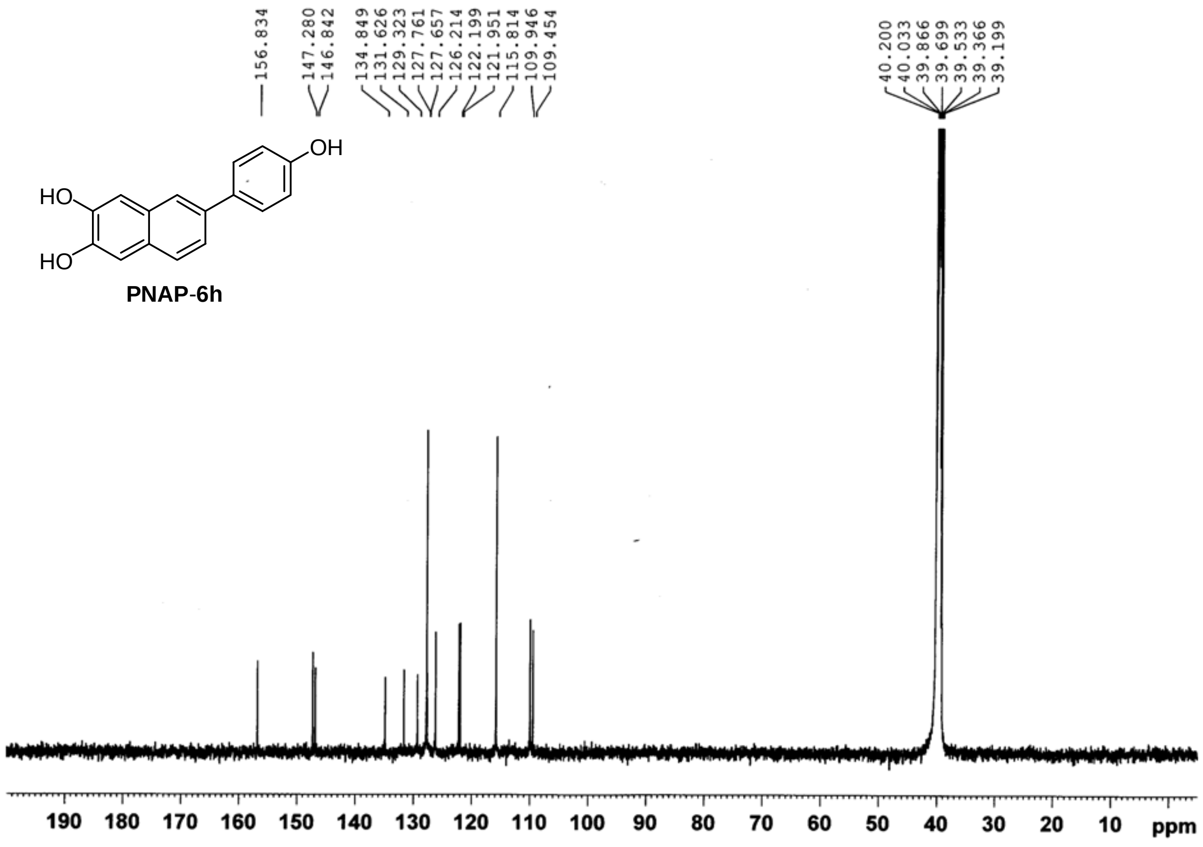

Figure E. ^{1}H and ^{13}C NMR spectra of PNAP-6h.

^{1}H NMR (500MHz, DMSO-d_6)

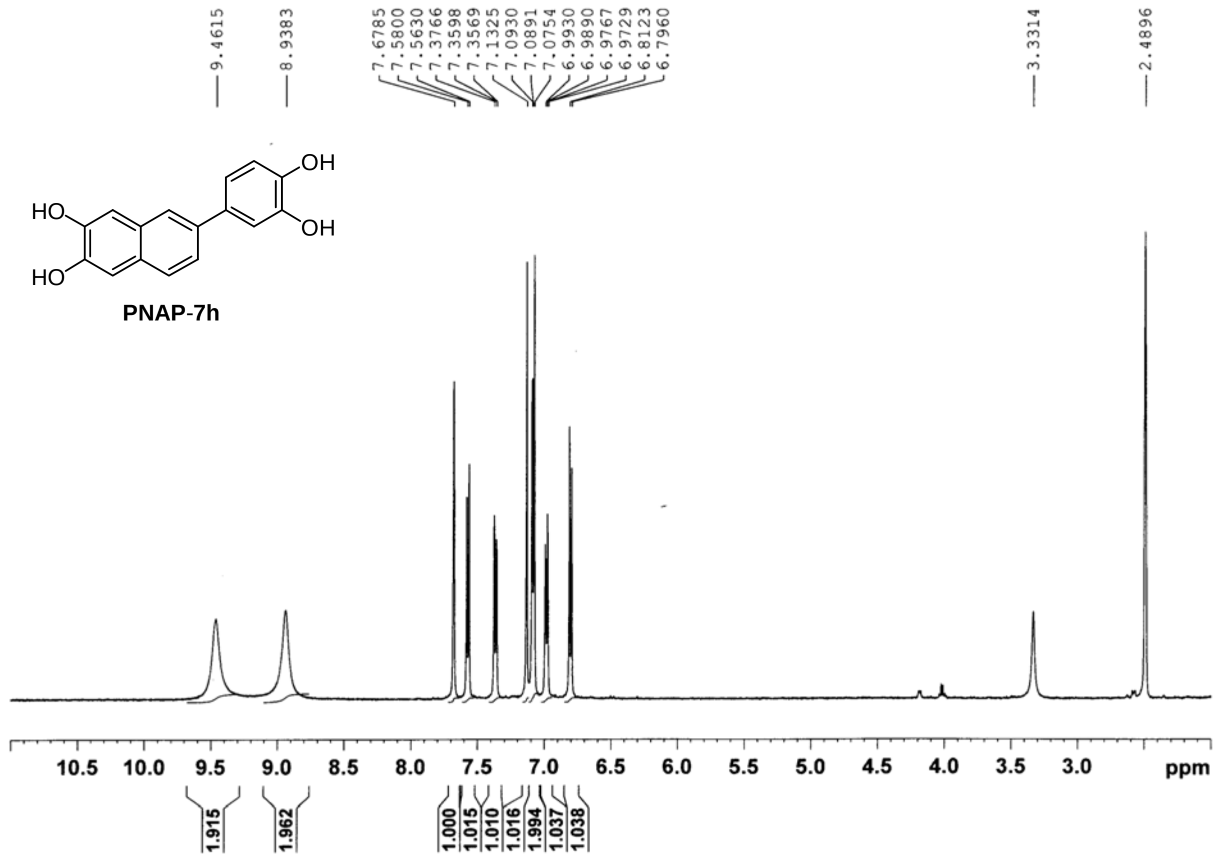

^{13}C NMR (125MHz, DMSO-d_6)

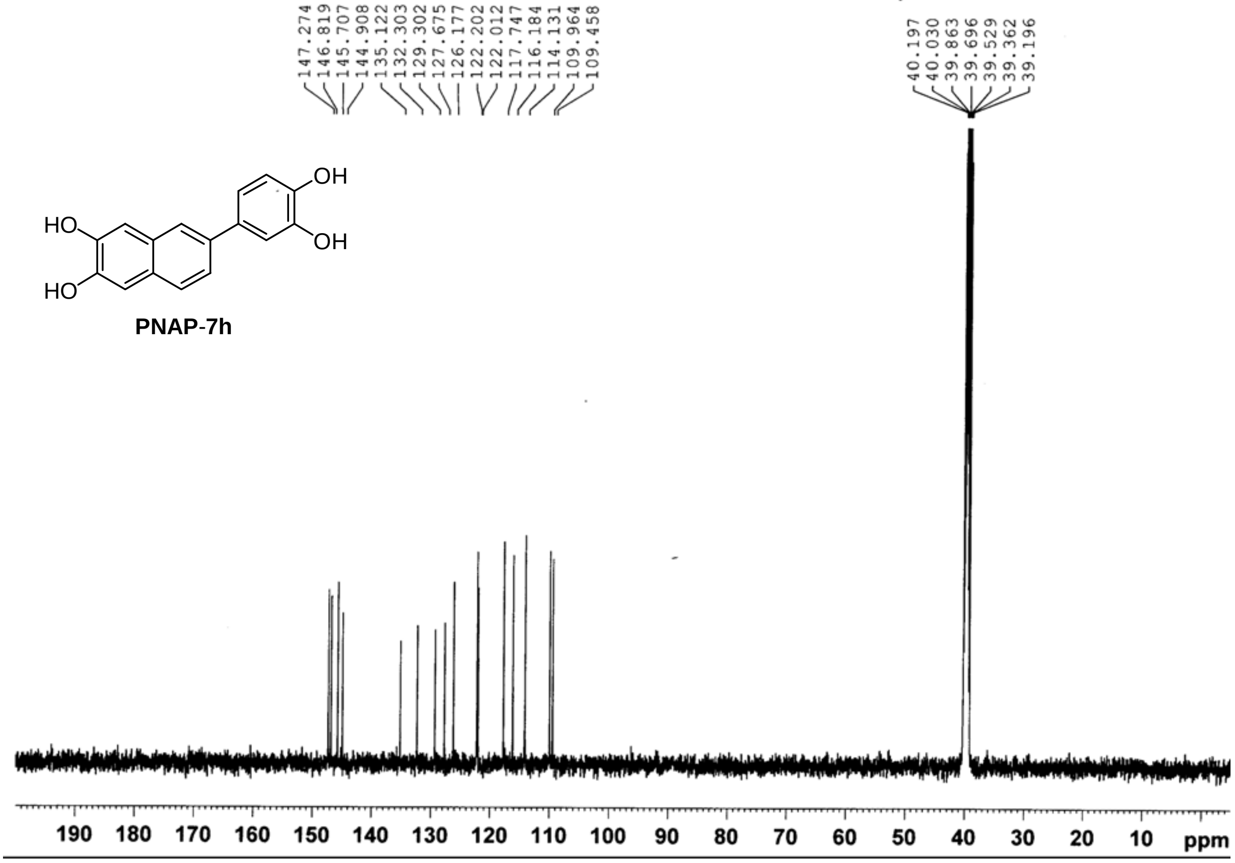

Figure F. ^{1}H and ^{13}C NMR spectra of PNAP-7h.

^{1}H NMR (500MHz, $CDCl_3$)

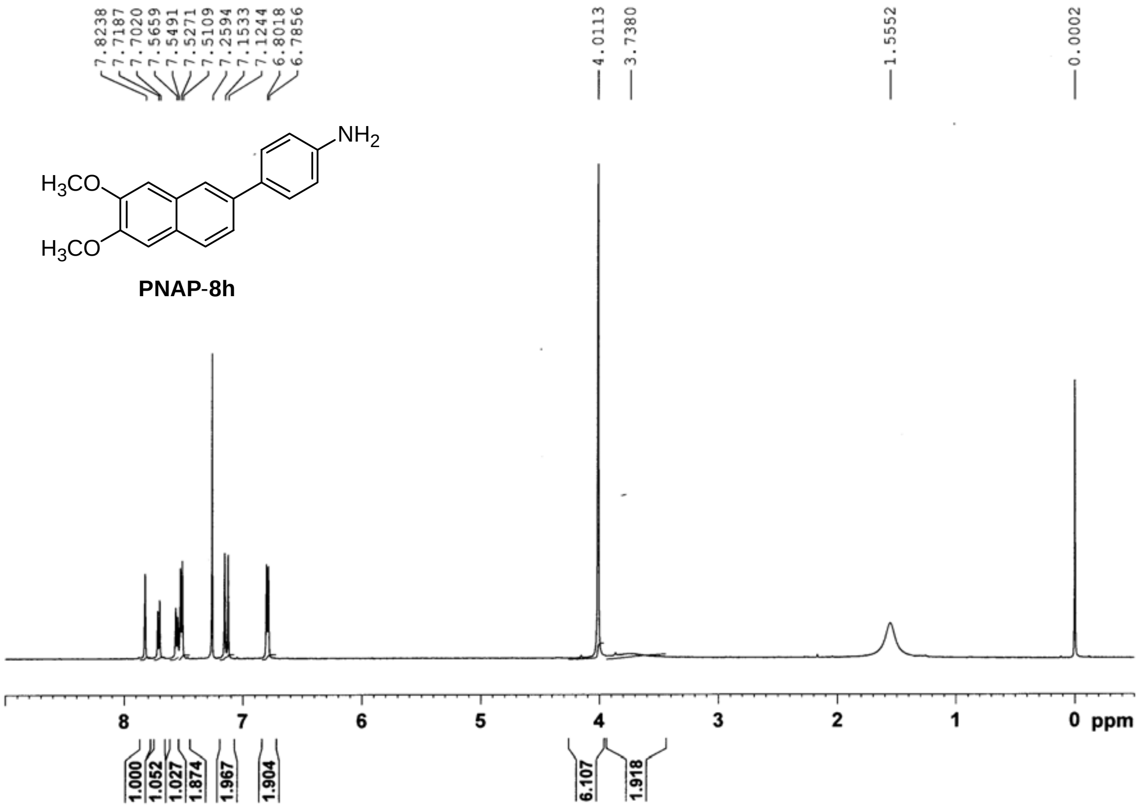

^{13}C NMR (125MHz, $CDCl_3$)

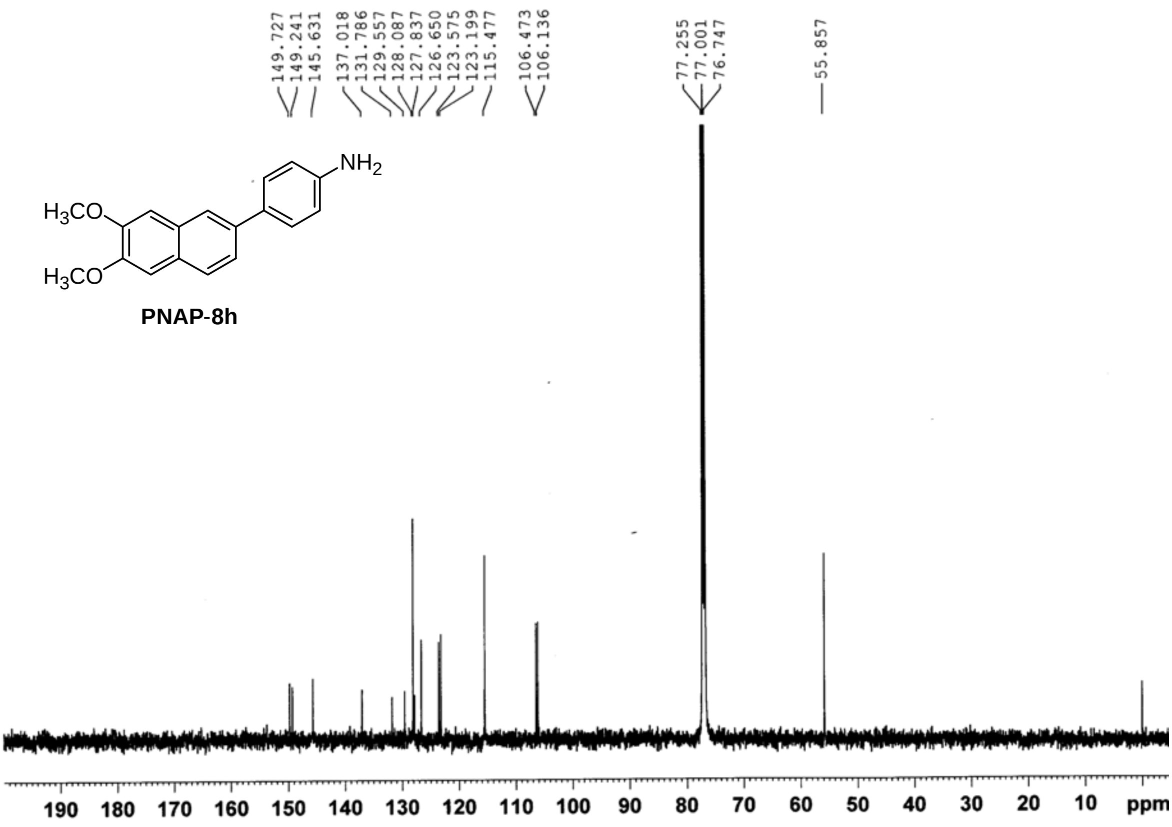

Figure G. ^{1}H and ^{13}C NMR spectra of PNAP-8h.
